# A patient‐centred conceptual model of nocturnal scratch and its impact in atopic dermatitis: A mixed‐methods study supporting the development of novel digital measurements

**DOI:** 10.1002/ski2.262

**Published:** 2023-07-01

**Authors:** Lucia Cesnakova, Keith Meadows, Stefan Avey, Judy Barrett, Brian Calimlim, Meenakshi Chatterjee, Sandra Goss, Katelyn R. Keyloun, Jérémy Lambert, Carrie A. Northcott, Francesco Patalano, Doina Sirbu, Wendy Smith Begolka, Nadia Thyssen, Sylvain Zorman, Jennifer C. Goldsack

**Affiliations:** ^1^ Digital Medicine Society (DiMe) Boston Massachusetts USA; ^2^ Health Outcomes Insights Ltd. Faringdon Oxfordshire UK; ^3^ Janssen Research & Development LLC Raritan New Jersey USA; ^4^ AbbVie, Inc. North Chicago Illinois USA; ^5^ UCB Pharma Brussels Belgium; ^6^ Pfizer, Inc. Cambridge Massachusetts USA; ^7^ Novartis Pharma AG Basel Switzerland; ^8^ National Eczema Association Novato California USA; ^9^ ActiGraph LLC Pensacola Flordia USA

## Abstract

**Background:**

Emerging digital measures and clinical outcome assessments (COAs) leveraging digital health technologies (DHTs) could address the need for objective, quantitative measures of symptoms of atopic dermatitis (AD), such as nocturnal scratching. Development of such measures needs to be supported by evidence reflecting meaningfulness to patients.

**Objectives:**

To assess nocturnal scratching as a concept of interest associated with meaningful aspects of health of patients with AD (adults and children); and to explore patient‐centred considerations for novel COAs measuring nocturnal scratch using DHTs.

**Methods:**

Phase 1 evaluated disease impacts on everyday life and the lived experience with nocturnal scratching through qualitative interviews of AD patients and caregivers. Phase 2 deployed a quantitative survey to a sample of AD patients as well as caregivers.

**Results:**

Four cohorts with various AD severity levels participated in Phase 1: (1) adults with AD (*n* = 15), (2) their caregivers/spouses/partners (*n* = 6), (3) children with AD (*n* = 14), and (4) their adult caregivers (*n* = 14). Findings were used to develop a conceptual model for nocturnal scratching as a potential concept of interest. The Phase 2 survey was completed by 1349 of 27640 invited adults with AD and caregivers of children with AD. The most burdensome aspects of AD reported were itchy skin and scratching. Overall, ∼65% of participants reported nocturnal scratching ≥1 day/week, resulting in ∼1–1.4 h of sleep lost per night. In all, 85%–91% of respondents considered it at least somewhat valuable that a treatment reduces night‐time scratching. About 50% reported willingness to use technology to this end and ∼25% were unsure.

**Conclusion:**

Our results represented by the conceptual model confirm that nocturnal scratch is a concept of interest related to meaningful aspects of health for patients with AD and therefore is worth being captured as a distinct outcome for clinical and research purposes. DHTs are suitable tools presenting an important measurement opportunity to assess and evaluate occurrence, frequency, and other parameters of nocturnal scratching as a disease biomarker or COA of treatment efficacy.

## Graphical abstract

Itch and sleep disturbances are two key symptoms associated with Atopic Dermatitis. Existing clinical outcome assessments (COAs) of assessing scratching in AD have several limitations. The development of novel measures and COAs, including those using digital health technologies, must be supported by evidence reflecting meaningfulness to patients. This work uses qualitative interviews with and survey of patients with AD and their caregivers to identify aspects of life affected by AD, as well as areas impacted by their nocturnal scratching. The results are captured in a conceptual model describing the meaningful aspects of health connected to nocturnal scratching, supporting development of novel COAs capturing this phenomenon.

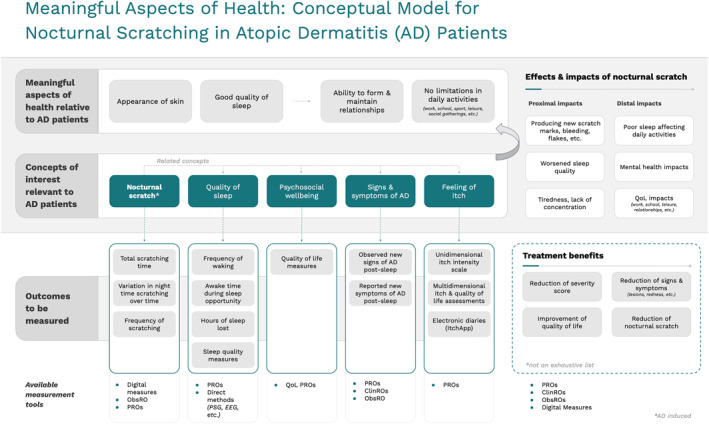

**What is already known about this topic**?

- Itch and sleep disturbances are two key symptoms associated with Atopic Dermatitis (AD). Itch, a sensation, is often used interchangeably with the action of scratching, which is an independent and also important symptom of AD.
- Existing clinical outcome assessments (COAs) of assessing scratching in AD have several limitations.
- The development of novel measures and COAs, including those using digital health technologies, must be supported by evidence reflecting meaningfulness to patients.

**What does this study add?**

- Qualitative interviews with patients with AD and their caregivers identified aspects of life affected by AD, as well as areas impacted by their nocturnal scratching.
- We developed a conceptual model for nocturnal scratching as a concept of interest, confirming the connection of nocturnal scratching with related concepts and meaningful aspects of health for AD patients.
- A quantitative survey explored details of experience, burden, and inclination to measurement of nocturnal scratching.

**What are the clinical implications of this work?**

- Meaningful aspects of health to AD patients – both adults and children – can define nocturnal scratching as a distinct outcome for both healthcare and research purposes.
- Measuring nocturnal scratching offers a quantitative method to evaluate the disease course and impact of therapeutic interventions.
- Novel assessments that leverage digital health technologies could help address the need for longitudinal, objective, quantitative measures of and key aspects of AD, such as nocturnal scratching.

## 1 INTRODUCTION

Atopic dermatitis (AD) is a chronic inflammatory skin disease with a prevalence of ∼17%–23% in developed countries, affecting both adults and children, as well as their partners and caregivers.^1^ Clinical features of AD include increased skin redness, thickness, lichenification and pruritus (itch), a near‐universal symptom^2^ that carries the greatest burden for skin diseases.^3^, ^4^ Itch has been associated with significant negative effects on patients' mood, sleep, functioning, and overall quality of life.^5^

Itch is complex to characterise, as it is a sensation composed of a variety of factors. The primary physical behaviour often accompanying itch is scratching.^6^ Scratching can damage the epidermal skin barrier, leading to allergen and bacterial infiltrations and inflammatory responses. It also leads to sensory neuron excitation and neural signalling, resulting in the feeling of itchiness. This has been termed the “itch‐scratch‐cycle” in AD, described as a complex pain‐like sensation (itch) with a reflex‐like response (scratching).^7^ These aspects can be also disassociated – a patient can scratch without being itchy, and feel itchy without scratching. Scratching has physical consequences, and also negatively affects the patient's quality of life.^5^ It is hypothesised to be more frequent in the evening and during sleep due to several mechanisms, including the disinhibited unconscious scratch response, circadian skin temperature variations, and increased trans‐epidermal water losses.^8^, ^9^ Because of their linkage, the terms of “itch” (a sensation) and “scratch” (an action) are often used interchangeably; however, they are two separate aspects of AD. To accurately capture endpoints meaningful to patients with AD, it is important to capture both components.

Existing clinical outcome assessments (COAs) of itch and scratch are based on clinician and patient reports.^10^ Physicians can assess scratching indirectly by observations of excoriations on the skin; however, this captures only visible markings and is challenging to quantify. Although patient‐reported outcomes are currently the only assessments that can be used to measure itch and scratching directly, they provide only one dimension to disease severity and quality‐of‐life assessments; can be subject to recall bias; cannot capture all incidences of scratching separate from itching; and may be difficult to use outside the clinic for capturing real‐time symptom fluctuation or continuous impacts of AD. The current gold standard for measuring nocturnal scratching, videography, likewise has several limitations: often it is performed in a sleep laboratory, where the patient is in unfamiliar surroundings not representative of their usual situation. Different bedding may itself induce flares due to certain materials and equipment and scoring mechanisms can vary. Videography methods are time‐consuming and expensive, generating only a “snapshot in time” of the patient's symptoms through subjective human‐rater annotation of the video. Such manual evaluation is tedious, often obstructed by use of blankets or bedding, and thus infeasible for the use in clinical care or large clinical trials with hundreds of patients. Thus there is a strong need to develop a quantitative measure capable of assessing and monitoring nocturnal scratching in patients' everyday lives. This would complement current patient‐ and clinician‐reported outcomes with a new dimension of information—direct measurement of patients' behaviour. Such a measure would find applications both as a disease biomarker and as a COA of treatment efficacy.

Digital health technologies (DHTs) could answer the need for an additional tool that adds an improved, precise, continuous objective measurement of nocturnal scratching. They can passively collect movement data, and process them algoritmically to quantify scratching.^11^, ^12^ The rise of DHTs and their use in healthcare and research has created the need for frameworks demonstrating their value. One published framework states that a concept of interest —a symptom or other specific aspect that can be practically measured—must be rooted in a meaningful aspect of health that matters to patients.^13^ Regulators have also encouraged stakeholders to prioritise patient needs and perspectives when developing new products, measures, and interventions.^14^ Despite the adoption of these frameworks, they rarely have been applied in research supporting how nocturnal scratch fits into existing conceptual AD models, its impacts, and measurement considerations.

In this study we used a mixed‐methods research approach to determine: (1) whether nocturnal scratch is a concept of interest that relates to meaningful aspects of health of patients with AD, including its various impacts, and (2) patient‐centred considerations for developing a novel COA using DHTs for clinical care and research. The goal of this research is to establish a novel patient‐centered evidence‐supported DHT‐based measurement solution for clinical research and care delivery.

## 2 PATIENTS AND METHODS

### 2.1 Study design and patients

A prospective mixed‐methods study was conducted in two phases. In Phase 1, we conducted individual qualitative concept‐elicitation interviews with participants with AD and their caregivers/partners/spouses, with the goal of concept saturation in all cohorts. We then drafted a quantitative patient survey and conducted cognitive testing interviews to ensure that the items were comprehensible and appropriately worded. Phase 2 consisted of administering the tested and finalised survey to a large population of participants with AD or their caregivers (in the case of children). The study protocol was approved by Solutions IRB, Yarnell, Arizona USA (approval number 2021/11/30), and patients (or adult caregivers of children with AD) provided informed consent (assent in the case of children) that was electronically recorded.

Patients and their caregivers/partners/spouses were eligible for inclusion in the study if they had US residency, a self‐reported diagnosis of AD or were a caregiver/partner/spouse of an adult or child with AD, an overall itch score ≥1 on Pruritus Numeric Rating Scale (pNRS) for adult or child patients,^15^ the ability to perform study assessments in English, and (for Phase 1) the ability to provide informed consent (assent in the case of patients <18 years old) to be interviewed and have the interview audio‐recorded. Participants were excluded for the presence of a skin condition other than AD, overall pNRS score of 0, or (for Phase 1) refusal to be audio‐recorded for transcript analysis.

We partnered with three US‐based patient advocacy groups—the National Eczema Association, Global Parents for Eczema Research, and the Asthma and Allergy Foundation of America—to solicit candidate participants for the one‐on‐one interviews. These groups advertised the study through their regular communications (newsletters, emails), and if members expressed interest, we sent them an invitation to undergo screening and provide informed consent.

### 2.2 Phase 1: Qualitative interviews

The goal was to recruit a maximum of 60 participants among four cohorts (15 per cohort): (1) adults with AD ≥18 years old (“Adults”), (2) adult caregivers/spouses/partners of adults with AD (“Partners”), (3) children 7–17 years old with AD (“Children”), and (4) adult primary caregivers of a child with AD (“Parents”). These sample sizes were deemed sufficient to achieve concept saturation, based on expert recommendation. Within each cohort, the goal was to have an even distribution of reported itch severities based on the pNRS: mild (<4), moderate (– <7), or severe/very severe pruritus (≥7). Recruitment of participants was monitored weekly, and was adjusted to ensure that age, sex, ethnicity, and itch severity levels of the final recruited cohorts were diversely represented. If the quota for a specific category of patient was fulfilled, additional candidates were not enrolled in that category.

In the interviews, Adults were paired with their Partners, if they had one. If not, then an Adult could be interviewed on their own. The second set of interviewed pairs consisted of a Child plus their Parent. Participants were reimbursed with a $200 USD Amazon gift card per pair. If an Adult did not have a Partner, they received a single $200 USD Amazon gift card on their own.

Appendices S1–S4 show the questions asked to participants. They were deemed appropriate in terms of readability and understandability for the adults or children (seventh‐grade reading level for all adult participants, fifth‐grade reading level for child participants). Participant interviews were conducted virtually by Health Outcome Insights Ltd. via Zoom using a semi‐structured topic guide within a timeframe of 60 min. All interviews were carried out with audio only (no video), and were recorded with participant consent. Anonymised audio recordings were transcribed verbatim by a professional transcribing agency.

Initial analysis of the transcripts for the four cohorts consisted of a full reading of each transcript by one expert rater (K.M.). Steps taken to analyse these data included manual compilation, organization, classification, and interpretation of the concepts. Transcripts were then provisionally coded based on the Framework to Guide the Selection and Development of Digital Measures of Health^13^ and FDA guidelines for patient‐centric drug development.^16^ Transcripts were then second‐level coded as sub‐elements of the text.^17^ A sample of verbatim quotations was selected from each of the cohort transcriptions to provide evidence of the interpretation of the data presented.

### 2.3 Development of conceptual model

The findings from the initial interviews were used to develop a conceptual model with a focus on nocturnal scratch as a concept of interest. This model represents how nocturnal scratching relates to other reported concepts and meaningful aspects of health relevant to AD patients. The model, based on a published “Measures that Matter” framework,^13^ also defined possible outcomes that would meaningfully capture aspects of nocturnal scratching.

### 2.4 Cognitive testing interviews

Before administration of the quantitative survey (Phase 2), we performed cognitive testing of the draft quantitative survey to ensure comprehension and question wording, retrieval of information, suitability of response options, and resolution of other found problems (e.g., question order). Cognitive testing was performed with the target populations of the survey. The individual participants were reimbursed after the interview with a $80 Amazon gift card.

Cognitive interviews were conducted by Health Outcomes Insights Ltd., over Zoom, with audio on and video off. Respondents were renamed to “Respondent”. Audio recordings were transcribed verbatim and coded as for the initial interviews.

The participants were either Adults or Parents who were not part of either the qualitative or quantitative part of the research. Participants were asked to read each question of the survey displayed on the screen. After reading each question, the participant was asked several open‐ended questions covering the issues listed above. Appendices S5 and S6 list the cognitive test questions and prompts for Adults and Parents, respectively. Issues of comprehension, recall etc. were reported together with expert recommendations for resolution.

### 2.5 Phase 2: Quantitative survey

For Phase 2, we conducted a single cross‐sectional survey, with the goal of reaching 1500 Adults and 1500 Parents that would be able to participate. We anticipated that ∼300 participants from each cohort would complete the survey. The target numbers were estimated based on the estimated population of AD patients in the digital survey platform used. The survey was conducted virtually on the Evidation consumer health and research platform (https://my.evidation.com/, Evidation Health, Inc., San Mateo, CA, USA), and participants could complete all study tasks at home.

Evidation is an online platform where people can connect their digital health tools and participate in research studies. Evidation members agree to being contacted with study opportunities when they create an account. Based on answers to already‐permissioned information from Evidation members (e.g., self‐reported AD), platform members were recruited via targeted emails and postings, offering an opportunity for them to participate in the survey. Potential participants were asked to complete an online screener to assess eligibility. If they satisfied all of the inclusion/exclusion criteria, they were first notified about how their survey responses would be used for research purposes through a Data Usage and Permissions Agreement, and then asked to complete the survey. Recruitment was planned to be monitored weekly to ensure a diverse population; however, the target recruitment was reached and enrolment closed within a week from study launch. Participants received 500 Evidation Points for completion of the survey. A level of 10000 Evidation Points can be exchanged for $10, which the member can keep or donate to a charitable organization.

The first part of the survey asked participants to rate the identified aspects and symptoms of AD experienced during the previous 2 weeks according to their frequency, importance, and level of bother on separate scales. This section also included the Patient‐Oriented Eczema Measure (POEM), a validated instrument used in AD research. The final POEM score enabled evaluation of AD severity in the previous week.^18^ The second part of the survey focused on the frequency, importance, level of bother, and effects on daily life caused by nocturnal scratching, and opinions about using digital technologies to measure this symptom. Appendices S7 and S8 list the questions asked of Adults and Parents, respectively.

### 2.6 Statistical analysis

Descriptive statistics were tabulated for demographic and disease characteristics for both phases of the study. For the quantitative survey, continuous variables were described by the number of observations and means with standard deviations. Categorical variables were summarised as frequencies and percentages. Only fully completed surveys were included in the analysis; therefore, missing data (from partly completed surveys) were not reported.

## 3 RESULTS

### 3.1 Phase 1: Qualitative interviews

**FIGURE 1:**
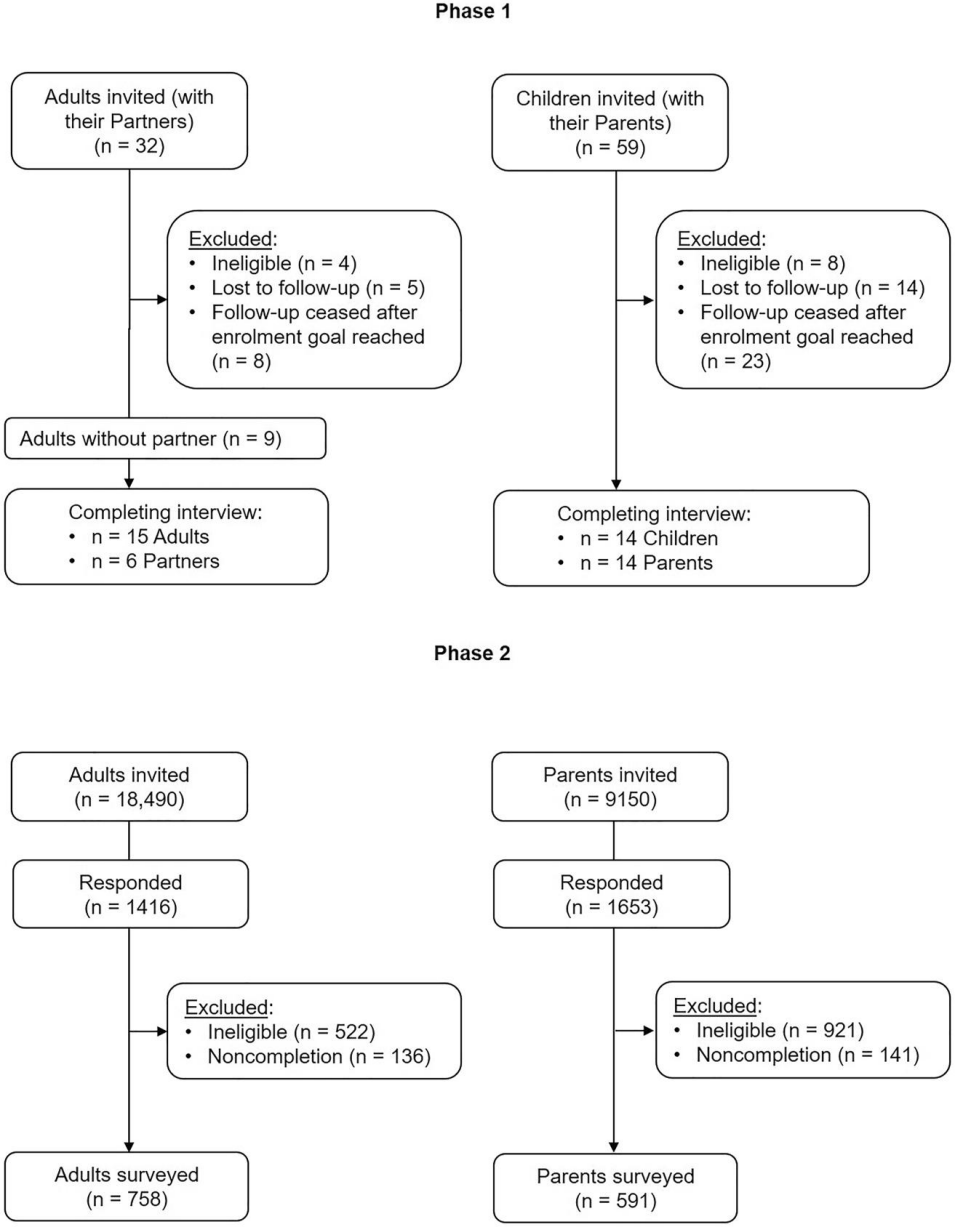
Disposition of participants and the process of enrolment in the study.

From 11 January 2022 to 8 February 2022, we invited a total of 32 Adults with their Partners (if they had one) and 59 Parents with their Children to participate (Figure 1). After exclusions, we conducted qualitative semi‐structured interviews with 15 Adults, 6 Partners, 14 Children, and their 14 Parents (described in detail in Table 1). The cohorts were diverse in terms of age, sex, and reported itch severity. Nine of the Adults did not have a Partner to be interviewed, and all of the 6 who *were* interviewed were male. Conversely, a majority of the interviewed Parents were female (79%).

**TABLE 1:** Demographic and disease characteristics of the participants with atopic dermatitis and their partner/caregivers who underwent qualitative interviews.

Adults with atopic dermatitis				Partners of adults		Children with atopic dermatitis				Parents of children	
ID	Sex	Age range (years)	Self–reported itch severity	Sex	Age range (years)	ID	Sex	Age range (years)	Self–reported itch severity	Sex	Age range (years)
ID1201	Female	25–34	Moderate	Male	25–34	ID3401	Female	6–9	Moderate	Female	35–50
ID1202	Female	18–24	Mild	—	—	ID3402	Male	6–9	Mild	Female	35–50
ID1203	Female	35–50	Moderate	Male	35–50	ID3403	Male	6–9	Moderate	Female	35–50
ID1204	Female	35–50	Moderate	—	—	ID3404	Male	6–9	Moderate	Female	35–50
ID1205	Female	25–34	Severe	Male	25–34	ID3405	Male	6–9	Mild	Female	35–50
ID1206	Male	35–50	Mild	—	—	ID3406	Male	10–12	Severe	Female	35–50
ID1207	Female	18–24	Mild	—	—	ID3407	Female	6–9	Moderate	Female	51–64
ID1208	Male	18–24	Mild	—	—	ID3408	Male	13–17	Moderate	Female	35–50
ID1209	Female	35–50	Moderate	Male	35–50	ID3409	Female	13–17	Severe	Female	35–50
ID1210	Female	25–34	Severe	—	—	ID3410	Female	13–17	Moderate	Female	35–50
ID1211	Female	18–24	Mild	—	—	ID3411	Female	13–17	Severe	Male	25–34
ID1212	Female	25–34	Severe	—	—	ID3412	Male	13–17	Severe	Male	25–34
ID1213	Female	18–24	Severe	Male	35–50	ID3414	Male	13–17	Severe	Male	25–34
ID1214	Female	35–50	Moderate	—	—	ID3416	Male	10–12	Mild	Female	35–50
ID1215	Female	25–34	Severe	Male	25–34	—	—	—	—	—	—

Scratching during sleep was reported by all interviewed participants in each cohort. The majority reported worsening of the itch sensation and urge to scratch in the evening/night, and observed signs of nocturnal scratching in the morning (skin flakes or cuts, blood, new scratch marks, etc.). Several participants reported observing these signs of nocturnal scratching in the morning without remembering the actual behaviour.

Nocturnal scratching affecting sleep was also a consistent finding across all cohorts. The reported impacts of nocturnal scratching included worsened skin condition, worsening of daily mood, feeling mentally and physically exhausted, lack of energy, and increased stress levels. Triggers for scratching for adults included fabrics, cosmetics, sweat, water contact, extremes of weather, and stress. Similarly, sweat was identified as a frequent trigger among Children.

Partners and Parents reported disturbed sleep because of their partner's/child's scratching, which affected their ability to function at work or perform daily activities. Though a small cohort in our study, the Partners also reported disturbed sleep by being woken up by their partner scratching or even being asked to check areas of skin of their partner. Parents also reported financial issues, difficulty with interpersonal relationships (child becoming distant), the child experiencing discrimination, worrying about the child, feeling unable to look after their child, and associated stress.

The importance of a treatment to reduce nocturnal scratching and other AD symptoms were consistently noted across all cohorts. Most participants reported willingness to use or wear a tool or a sensor for their own education and to support research into new treatments, although they also reported reservations about privacy, comfort, or wear location in relation to DHT tools.

Appendix S9 provides a representative collection of verbatim responses from the participants.

### 3.2 Conceptual model

Conceptual saturation of several factors—including sign/symptom concepts—were assessed from the post‐interview transcripts, and was achieved for Adults, Children, and Parents, indicating that no further interviews were necessary. Due to the limited number of Partner participants interviewed, concept saturation was not able to be determined.

**FIGURE 2:**
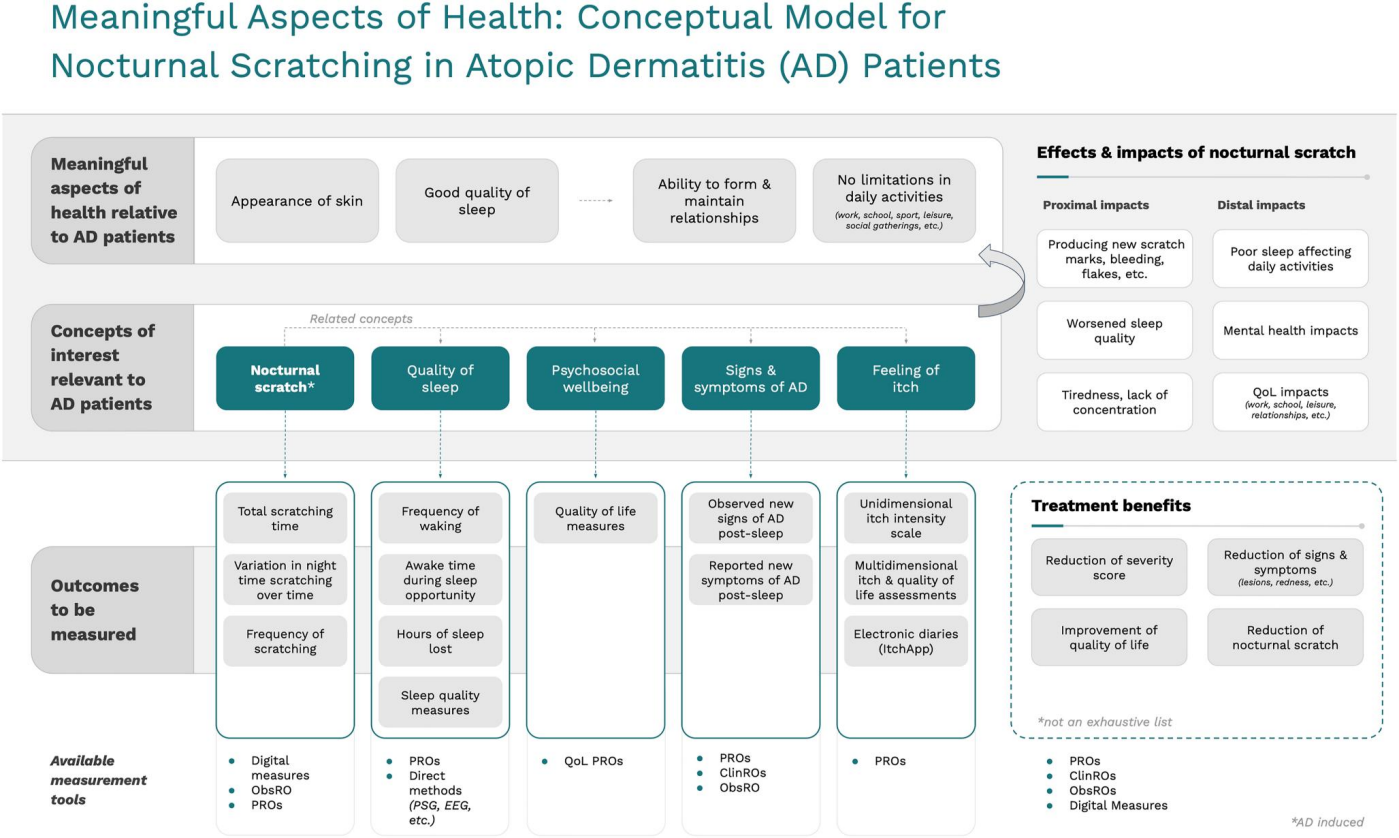
Proposed model for the interrelationships between nocturnal scratching and key factors, based on a thematic analysis of narratives from qualitative interviews of adult and child patients with atopic dermatitis and their primary adult caregivers.

Based on thematic analyses of the narratives from the qualitative interviews, we developed a conceptual model for the interrelationships between key factors and nocturnal scratching (Figure 2). For all cohorts, meaningful aspects of health related to AD included skin appearance, good quality of sleep, and (from these implicated) the ability to form and maintain relationships; and no limitations on daily activities. This model further informed the development of the quantitative survey administered in Phase 2 of the study.

### 3.3 Phase 2: Quantitative survey

From 25 to 29 April 2022, we conducted cognitive interviews to evaluate the draft survey items with Adults (*n* = 5) and Parents (*n* = 5). As a result, the survey content was deemed suitable, and only minor edits were made prior to finalisation. Between 10 and 15 June 2022, Evidation then sent an invitation to participate in the survey to 18490 adult members of the Evidation platform who self‐reported having AD, and to 9150 members who reported having a child with AD (Figure 1). Of the Adults, 1416 (7.7%) were screened, and 758 (4.0%) were eligible and completed the survey. Of the Parents, 1653 (18%) were screened, and 591 (6.5%) were eligible and completed the survey.

Demographic and disease characteristics of the respondents are shown in Table 2. In all, 90% of the Adults and 49% of the children (by the report of their surveyed Parent) were female, and, based on POEM score,^17^ 62% of Adults and 51% of children (by the report of their surveyed Parent) had at least moderate AD within the previous week (Table 2).

**TABLE 2:** Demographic and disease characteristics of adults with atopic dermatitis (AD) and caregivers of children with AD who responded to a quantitative survey and completed the questionnaire.

Characteristic, *n* (%)	Adults with AD	Caregiver for child with AD	Child with AD
	(*n* = 758)	(*n* = 591)	(*n* = 591)
Age of adult			
18–24 years	54 (7.1)	4 (0.7)	—
25–34 years	292 (38.5)	114 (19.3)	—
35–50 years	317 (41.8)	415 (70.2)	—
51–64 years	74 (9.8)	56 (9.5)	—
≥65 years	21 (2.8)	2 (0.3)	—
Age of the child			
≤5 years	—	—	126 (21.3)
6–9 years	—	—	159 (26.9)
10–12 years	—	—	114 (19.3)
13–17 years	—	—	192 (32.5)
Sex			
Female	685 (90.4)	497 (84.1)	289 (48.9)
Male	68 (9.0)	90 (15.2)	293 (49.6)
Prefer not to answer	2 (0.3)	4 (0.7)	5 (0.9)
Other	3 (0.4)	0	4 (0.7)
Race/ethnicity			
White	608 (80.2)	476 (80.5)	476 (80.5)
Asian	72 (9.5)	42 (7.1)	50 (8.5)
Black	54 (7.1)	49 (8.3)	66 (11.1)
Hispanic, Latino, or Spanish	53 (7.0)	47 (8.0)	64 (10.8)
American Indian or Alaska Native	9 (1.2)	5 (0.9)	11 (1.9)
Native Hawaiian or Pacific Islander	7 (0.9)	4 (0.7)	6 (1.0)
Other race/ethnicity	7 (0.9)	3 (0.5)	3 (0.5)
Prefer not to answer	7 (0.9)	7 (1.2)	7 (1.2)
Middle Eastern or Northern African	2 (0.3)	5 (0.9)	5 (0.9)
Self‐reported severity of atopic dermatitis^a^			
Clear	55 (7.3)	—	45 (7.6)
Mild	237 (31.3)	—	246 (41.6)
Moderate	343 (45.3)	—	234 (39.6)
Severe	106 (14.0)	—	54 (9.1)
Very severe	17 (2.2)	—	12 (2.0)

^a^ Based on the Patient‐Oriented Eczema Measure (POEM) tool.^18^ The data are presented as *n* (%).

**FIGURE 3:**
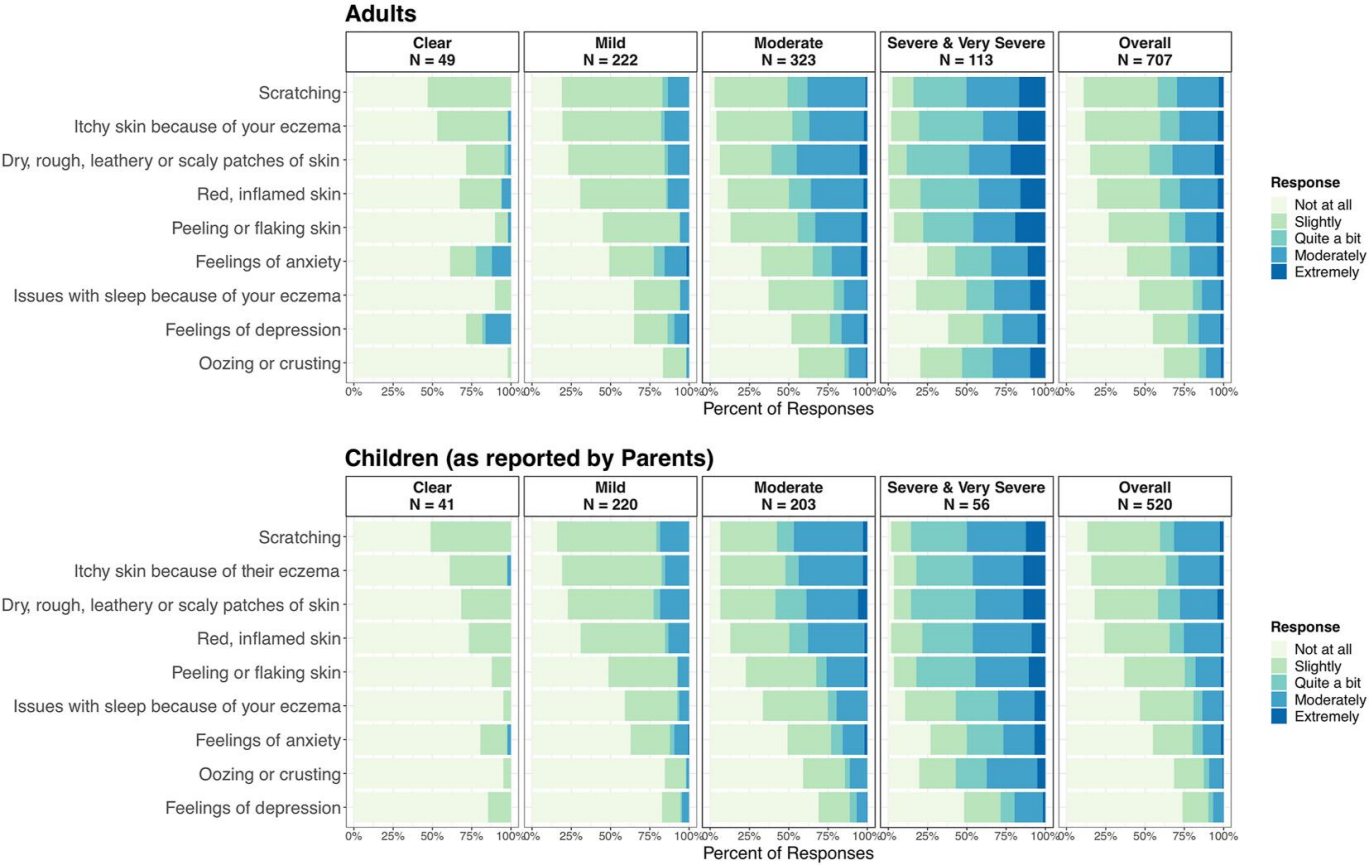
Responses to Phase 2 survey question asking: “During the past two weeks, how burdensome did you find (Adults) / what is your impression of how burdensome your child found (Parents on behalf of Children) the following?” The results are shown per severity of AD (based on POEM score) and overall. Responses are ordered separately for Adults and Children by percentage of participants overall. Data are displayed for 707 (Adults) / 520 (Parents on behalf of Children) participants who correctly identified itch as a sensation in the test question (see Appendices S7, S8).

We provided descriptions of itch and scratching within the survey to ensure clarity on the terminology. A majority of Adults (93%) and Parents (88%) were able to identify the appropriate terminology regarding itch and scratch in a test question (see Appendices S7, S8). As the severity of AD increased, there was an increased bothersomeness, intensity, and frequency observed in all surveyed symptoms and effects. As shown in Figure 3, the most burdensome AD symptoms reported by Adults were itchy skin; dry, rough, leathery, or scaly patches of skin; and scratching. This was consistent with the reports from Parents. Resting and sleeping were the activities most limited by AD for both cohorts.

In all, 70% of Adults and 75% of Parents were aware of nocturnal scratching, with 67% and 65%, respectively, reporting nocturnal scratching at least 1 night per week. The most often observed sign of nocturnal scratching was irritated or red skin in the morning, reported by 73% of Adults and 67% of Parents. Nocturnal scratching was reported to result in 1 h of sleep lost per night for Adults, 1.4 h per night for Children (as reported by Parents), and 1.2 h per night for Parents themselves. Disturbed sleep most often resulted in not feeling rested in the morning and feeling tired during the day (47% of Adults, 46% of Parents for 1 night or more in the previous 2 weeks), as well as not being able to focus (38% of Adults, 35% of Parents for 1 night or more in the previous 2 weeks).

The most‐used efforts to reduce or prevent nocturnal scratching included application of creams, lotions, or topical medications before sleep and showering before going to sleep. Majority of Adults (85%) and Parents (91%) ranked effectiveness of a treatment in reducing nocturnal scratching as important, with the goals of having better sleep in general and spending less total time scratching at night being considered most important.

The ability to measure nocturnal scratching was ranked as valuable by 70% of Adults and 80% of Parents, and 53% and 46%, respectively, indicated willingness to use technology for this purpose (25% and 28% were unsure whether they would or not). Respondents reported that they would use the data to help improve their AD in general, help them talk to their doctors about their AD, and help researchers learn more about AD. The areas of most concern regarding this use of technology included physical discomfort and interference with sleep. Detailed survey data supporting the above narrative are reported in Appendix S10.

## 4 DISCUSSION

In this mixed‐methods study, nocturnal scratching was shown to be a concept of interest for adults and children with AD that has a strong connection to meaningful aspects of their health and lives. In both the qualitative interviews and the quantitative survey, participants detailed the effects of the disease on activities in their daily life and on sleep, resulting in information that can be used to define meaningful outcomes for clinical practice and research.

In the development of the conceptual model, we report the findings using a framework to determine the aspects of lives that are most meaningful (Figure 2). This approach supported relationships among symptoms, behaviours, experiences and burden on living as well as their connections to relevant outcomes. Positioning nocturnal scratching in this framework established it as a potential target of novel COAs that can leverage DHTs in their assessment.

This study also explored the effects of nocturnal scratching on participants' lives. The impact of nocturnal scratching is substantive, with an average of 1 h of sleep lost during the night for adult and child patients as well as their caregivers. Given that regular nightly sleep consists of several cycles lasting ∼90–110 min each,^19^ AD and scratching‐related interference with sleep—even for short periods—likely have negative impacts on quality of sleep and rest. This was also seen in the transcripts, as the patients indicated not feeling rested and being unable to focus during the day due to night‐time scratching.

Within the survey it was noted that ∼50% of both adults and caregivers reported willingness to use digital technologies to measure nocturnal scratching, while 25% of adults and 28% of caregivers were unsure about whether they would be willing to use such technology. This highlights the potential of additional education regarding the use of DHT for medical uses, which may bring value in terms of their acceptance by patients. Based on our results, technology developers and manufacturers also should strongly consider the patient experience of using the technology (e.g., comfort of wear, potential skin irritation, sleep interference, potential duration and frequency of wear/use in research studies) and ensuring data privacy. This will be ensured through a growing body of evidence regarding the value of these DHT‐based measures in understanding and treating diseases.

Future research should address correlations between digitally measured nocturnal scratching and measures currently used in AD to understand more fully the relationship between them. However, given that they do not always capture concepts equivalent to scratching at night, this work may be challenging. Similarly, studies should assess whether improvements in digitally measured nocturnal scratching are correlated to improvements in both general and dermatology‐specific quality‐of‐life measures, either spontaneously or after appropriate therapeutic intervention. It would also be valuable to clinicians, patients, healthcare decision‐makers, and payers to determine the minimal important difference and whether improvements in digitally measured nocturnal scratching are associated with reduced overall healthcare spending and resource use. Future research should also address verification, analytic validation and clinical validation of a novel COA leveraging DHTs to measure nocturnal scratch. Finally, this novel outcome measure may aid research in other pruritic conditions that have substantial negative impact on patients' lives, such as psoriasis.^20^

This study has several limitations. A single expert rater analysed the interview transcripts. The surveyed participants were skewed heavily towards women. Although comparison of female and male participants' answers did not show significant differences, the results cannot be generalised to the whole population because of the skewed ratio of the represented sexes. Evidation is a virtual platform, so there was a bias towards people with higher education and who are potentially more technologically savvy. The data obtained was based on participants from the U.S.A., therefore may not be easily extrapolated to other regions of the word. The diagnosis and severity of AD were not confirmed by clinician assessment and was based on the patients reporting and POEM measure (Phase 2). The response rate to the quantitative survey was lower than desired, which may limit the generalisability of our findings. Larger and/or more diverse populations might have different perspectives on the effects of AD in their daily lives. Additional research is suggested to assess dynamic properties and more complex potential relations between the disease concepts.

## 5 CONCLUSION

In this mixed‐methods study, we identified nocturnal scratching as a concept of interest that is important to patients with AD and connected to meaningful aspects of their lives. The extensive patient input collected on the burden and experience of this symptom supports it as an important outcome measure in clinical research. We believe this study serves as a foundation for future research to develop novel clinical outcome assessments of nocturnal scratch, patient acceptance and education, and development of specific DHTs.

## CONFLICT OF INTEREST STATEMENT

Lucia Cesnakova and Jennifer C. Goldsack are employees of the Digital Medicine Society, the sponsor of this study. The other authors are employees of their respective organizations. Carrie A. Northcott, Doina Sirbu and Jérémy Lambert are shareholders of their respective organizations.

## AUTHOR CONTRIBUTIONS

**Lucia Cesnakova**: Conceptualization (equal); Data curation (equal); formal analysis (equal); investigation (equal); Methodology (equal); project administration; lead, supervision (equal); visualization (equal); writing—original draft (equal); writing—review & editing (equal). **Keith Meadows**: Data curation (equal); formal analysis (equal); investigation (equal); methodology (equal); writing—review & editing (equal). **Stefan Avey**: Conceptualization (equal); data curation (equal); methodology (equal); visualization (equal); writing—review & editing (equal). **Judy Barrett**: Investigation (equal); writing—review & editing (equal). **Brian Calimlim**: Conceptualization (equal); methodology (equal); writing—review & editing (equal). **Meenakshi Chatterjee**: Conceptualization (equal); methodology (equal); writing—review & editing (equal). **Sandra Goss**: Conceptualization (equal); methodology (equal); writing—review & editing (equal). **Katelyn R. Keyloun**: Conceptualization (equal); methodology (equal); writing—review & editing (equal). **Jérémy Lambert**: Conceptualization (equal); methodology (equal); writing—review & editing (equal). **Carrie A. Northcott**: Conceptualization (equal); methodology (equal); writing—review & editing (equal). **Francesco Patalano**: Conceptualization (equal); methodology (equal); writing—review & editing (equal). **Doina Sirbu**: Conceptualization (equal); methodology (equal); writing—review & editing (equal). **Wendy Smith Begolka**: Conceptualization (equal); methodology (equal); resources (equal); writing—review & editing (equal). **Nadia Thyssen**: Conceptualization (equal); methodology (equal); writing—review & editing (equal). **Sylvain Zorman**: Conceptualization (equal); methodology (equal); writing—review & editing (equal). **Jennifer C. Goldsack**: Conceptualization (equal); methodology (equal); project administration (equal); resources (equal); supervision (equal); writing—review & editing (equal).

## ETHICS STATEMENT

The study protocol was approved by Solutions IRB, Yarnell, Arizona USA (approval number 2021/11/30).

## Supporting information

Supporting Information S1

## Data Availability

The complete data supporting the results in this paper are archived on the Digital Society Medicine (DiMe) virtual drive, which are available on request by qualified researchers. A subset of curated quantitative data is available publicly at https://dimesociety.org/access-resources/digital-measures-nocturnal-scratch/#research.
