# Supplementary material for: A patient‐centred conceptual model of nocturnal scratch and its impact in atopic dermatitis: A mixed‐methods study supporting the development of novel digital measurements
Source: Skin Health Dis. 2023 Jul 1;3(5):e262. doi: 10.1002/ski2.262 (PMC10549806; doi:10.1002/ski2.262)

## **Appendix S1. Qualitative Interview Questions for**: Adults diagnosed with atopic dermatitis (18 years and older)

**Introduction to the interview**

“Welcome to this interview that is a part of the *Study to identify meaningful aspects of health connected to the symptom of nocturnal scratching in patients with atopic dermatitis.* My name is __________ and I will perform the interview with you today.

Please allow me to remind you that this interview is audio recorded. You can decide to withdraw at any time during our talk for any reason. In such case, just tell me if you wish not to continue.

In this interview we will focus on your life and everyday experience with atopic dermatitis, otherwise known as eczema. We will also look more into your eczema and its symptoms. We will ask about your **night-time symptoms**. The night-time symptoms happen from when you get into bed until you wake up. Therefore, by saying “night-time symptoms,” we actually mean “symptoms during the time when you intend to/would like to sleep, and the time when you do actually sleep, until you get out of bed.”

We will also use terms **itch** and **scratch**, and by these we don’t mean the same thing. We will refer to “itch” as the sensation that you have, the feeling of itchiness you perceive. We’ll use the term “scratch” as the action of scratching, meaning something you can do to your skin, the action. It is just like when you are hungry, which is a feeling, and when you eat, which is an action. It also doesn’t necessarily mean you are using your fingers to do the scratching. It can be any physical action or motion on the skin or clothing. It’s important to realize that they can happen together, or they can happen on their own. For example, you feel itchy, so you start scratching; or you can feel itchy and not scratch, or you can scratch yourself without the feeling of itch.”

**Understanding of meaningful aspects of daily life with eczema**

**Introduction:** Let’s start with a few questions about your eczema in general.

1. Can you tell me about your eczema since the time you have been diagnosed?
   1. PROBE: Can you tell me more about that, or give me an example from a recent time?
2. Does eczema affect your everyday life? [If yes, PROBES:]
   1. PROBE:
      1. Can you tell me more about that, or give an example from a recent time?
      2. What is the most frustrating thing about your eczema? Why?
3. Is there anything you wish you could do, but due to your eczema you can’t, or don’t want to, do that activity? [If yes, PROBES:]
   1. Would you tell me more about the [activity]
      1. How does your condition prevent you from doing this?
      2. Would you do it if you haven’t had your condition? How often?
4. What symptoms have the biggest impact on your daily life?
   1. PROBES:
      1. Can you tell me more about that, or give an example from a recent time?
      2. What are some physical impacts your symptoms have on your life? What about emotional impacts? And are there any social impacts resulting from your eczema?
      3. What symptoms are the most frustrating?

**Focus on night-time scratch and expectations from treatment**

**Introduction:** Now we want to just focus on the symptom of night-time scratching.

1. Do you scratch after you go to bed or while you are sleeping? [If yes, PROBES:]
   1. How do you know you have scratched yourself after you go to bed or while you are sleeping?
      1. Have you ever found evidence of scratching in the morning (scratch marks, skin flakes, blood on sheets, etc.) even if you don’t remember scratching yourself? [If yes, PROBES:]
         1. What was the evidence you observed? Can you elaborate?
         2. Have you witnessed any of the following: skin redness, skin thickness, flaking, crusting or oozing? If yes, which?
         3. How often does this happen?
   2. How does the scratching differ between your awake time and your night-time? (by night we mean “the time when you want to sleep”)
      1. When is it most bothersome - daytime or night-time?
   3. Do you scratch after you go to bed, but before you fall asleep, in other words when you want to be sleeping?
      1. [If yes] How often does it happen?
   4. Do you think that scratching wakes you up from your sleep? [If yes] How often does it happen? How long does it take you to go back to sleep?
   5. Do you think it affects how well you sleep and whether you feel rested in the morning?
   6. Do you do anything special to prevent your scratching during sleep time? Can you give some examples?
   7. Does your night-time scratching vary across a longer period of time?
      1. If yes, are there nights when you scratch more, and nights when you scratch less?
      2. How does the scratching at night change when you have a flare-up or your disease gets worse? How?
   8. Does the night-time scratching leave any marks on your skin? [If yes] How often does it happen? How does this make you feel?
2. Is it important to you that a treatment for eczema would also lessen your overall scratching? [If yes, PROBES:]
   1. What would you consider an improvement in your night-time scratching?
      1. PROBES: Would it be less intense / less often / last shorter of the scratching episodes - or other?
   2. Do you think reducing your night-time scratching would improve your everyday life? [If yes, PROBES:]
      1. How?
      2. PROBES: What would get better about your days? What activities would be easier to do for you? How would you like to feel during the day?
   3. Do you think reducing your night-time scratching would improve your sleep? [If yes, PROBES:]
      1. How?
      2. PROBES: fall asleep faster, wake less often from your sleep, feel better rested in the morning - or other?
3. We understand it may be hard to tell the amount of scratching you have at night, or if it is getting better or worse. If there was a way to tell you about the amount of scratching you are experiencing at night, would this be valuable to you? [If yes, PROBES:]
   1. Would you be willing to wear a tool or sensor when you intend to sleep to capture this data?
      1. If yes, why? If no, why not?
   2. Would you be willing to use some kind of “room sensor” which could do this, something that would not be worn directly?
      1. If yes, why? If no, why not?

**Understanding of broader impact of scratching on patient’s life**

**Introduction:** Now we want to focus on what are some broader impacts of your night-time scratching on your life.

1. Does the night-time scratching (during the time you’d like to sleep) have any impact on your **sleep or the quality of your sleep**? [If yes, PROBES:]
   1. What are the impacts the night-time scratching (during the time you’d like to sleep) has on your sleep or the quality of your sleep?
      1. [to elaborate] Can you tell me more about that, or give an example from a recent time?
   2. What are the impacts on your sleep quantity (how much you sleep)? What about sleep quality (how well you sleep)?
2. Does the night-time scratching (during the time you’d like to sleep) have any impact on your **daily life**? [If yes, PROBES:]
   1. What are these impacts?
      1. [to elaborate] Can you tell me more about that, or give an example from a recent time?
   2. Is there anything that you find difficult to do during the day because of your night-time scratching?
   3. What do you consider the most bothersome aspects of your night-time scratching regarding your life during the day?
   4. How does night-time scratching compare to other symptoms you mentioned?
      1. Does scratching impact your daily life more or less versus other symptoms you mentioned?
3. (If the participant has indicated working or going to school currently) Does the night-time scratching (during the time you’d like to sleep) have any impact on your **work or school life**? [If yes, PROBES:]
   1. What are these impacts?
      1. [to elaborate] Can you tell me more about that, or give an example from a recent time?
4. Does night-time scratching (during the time you’d like to sleep) have any impact on your **social life**? [If yes, PROBES:]
   1. What are these impacts?
      1. [to elaborate] Can you tell me more about that, or give an example from a recent time?

**Closing the interview:**

Thank you for your time today. The audio recording will stop after we close this session.

## **Appendix S2. Qualitative Interview Questions** for: Adult caregivers of adult AD subject

**Introduction to the interview**

“Welcome to this interview that is a part of the *Study to identify meaningful aspects of health connected to the symptom of nocturnal scratching in patients with atopic dermatitis.* My name is __________ and I will perform the interview with you today.

Please allow me to remind you that this interview is audio recorded. You can decide to withdraw at any time during our talk for any reason. In such case, just tell me if you wish not to continue.

[establish relationship to the patient (caregiver/spouse/significant other/partner) for referencing throughout the interview]

In this interview we will focus on your and your [significant other’s] life and everyday experience with atopic dermatitis, otherwise known as eczema. We will also look more into your observations of their eczema and its symptoms. We will ask about their **night-time symptoms**. The night-time symptoms happen from when one gets into bed until one wakes up. Therefore, by saying “night-time symptoms”, we actually mean “symptoms during the time when someone intend to/would like to sleep, and the time when you do actually sleep, until you get out of bed”.

We will also use terms **itch** and **scratch**, and by these we don’t mean the same thing. We will refer to “itch” as the sensation, the feeling of itchiness. We’ll use the term “scratch” as the action, meaning something one can do to their skin. It is just like when you are hungry, this is a feeling or sensation, and when you eat, this is an action. It also doesn’t mean using scratching with the fingers or hand, it can be any physical action or motion on the skin or clothing. It’s important to realize that they can happen together, or they can happen on their own.”

**Understanding of meaningful aspects of daily life of a caregiver for an adult with eczema**

**Introduction:** Let’s start with asking a few questions about your [significant other’s] eczema in general

1. Can you tell me about the eczema of your [significant other] since the time **they** have been diagnosed?
   1. PROBE: Can you tell me more about that, or give me an example from a recent time?
2. Have you observed eczema impacting **their** daily life at all? [If yes, PROBES:]
   1. How?
      1. PROBE: Can you tell me more about that, or give me an example from a recent time?
   2. What do you think are the symptoms that have the biggest impact on **their** daily life?
3. Does their eczema have any impact on **your** daily life in any way? [If yes, PROBES:]
   1. What are these impacts?
   2. PROBE: Can you tell me more about that, or give an example from a recent time?
   3. What do you think are the symptoms that have the biggest impact on **your** daily life?
   4. What about their symptoms is the most challenging for you?

**Focus on nocturnal scratching and expectations from treatment**

**Introduction:** Now we want to just focus on the symptom of night-time scratching.

1. Have you observed that your [significant other] would scratch after they go to bed to go to sleep? [If yes, PROBES:]
   1. How did you observe this?
      1. Have you ever found evidence of scratching in the morning, (scratch marks, skin flakes, blood on sheets, etc.) even if you or [significant other] don’t remember scratching themselves?
      2. What was the evidence you observed? Can you elaborate?
      3. Have you witnessed any of the following: skin redness, skin thickness, flaking, crusting or oozing? If yes, which?
      4. How often does this happen?
   2. Does the scratching of **your [significant other]** during this time differ from scratching during their awake time? How?
   3. Does **your [significant other]** scratch after they go to bed, but before they fall asleep? [If yes] How often does it happen?
   4. Have you noticed scratching would wake **your [significant other]** from sleep? [If yes] How often?
      1. Does it wake **you** from sleep? [If yes] How often?
   5. Does **your [significant other’s]** night-time scratching vary across a longer period of time?
      1. If yes, are there nights when they scratch more, and nights when they scratch less?
      2. How does the scratching at night change when your [significant other] has a flare-up or their disease gets worse?
   6. Does the night-time scratching leave any marks on your [significant other’s] skin? [If yes] How often does it happen?
   7. What effects do you observe night-time scratching has on **your [significant other’s]** work or school life? Their social life?
2. Is it important to you that a treatment for eczema would also lessen the overall scratching of your [significant other]? [If yes, PROBES:]
   1. Is it important to you that a treatment would also lessen their night-time scratching?
      1. What would you consider an improvement in your [significant other’s] night-time scratching?
         1. PROBES: Would it be less intense / less often / last shorter of the scratching episodes - or other?
   2. Do you think reducing your [significant other’s] night-time scratching would improve **their everyday life**? [If yes, PROBES:]
      1. How?
   3. Do you think reducing your [significant other’s] night-time scratching would improve **their sleep**? [If yes, PROBES:]
      1. How?
      2. PROBES: fall asleep faster, wake less often from your sleep, feel better rested in the morning - or other?
   4. Do you think it would bring any improvements for **you** if your [significant other] has better nights less disturbed by their night-time scratching? [If yes, PROBES:]
      1. What would be those improvements?

**Understanding of broader impact of scratching on caregiver’s life**

**Introduction:** Now we want to focus on what are some broader impacts of your [significant other’s] night-time scratching on your life as a parent or caregiver.

1. Does the night-time scratching (during the time you’d like to sleep) **of your [significant other]** have any impact on **your sleep or the quality of your sleep**? [If yes, PROBES:]
   1. What are these impacts?
      1. [to elaborate] Can you tell me more about that, or give an example from a recent time?
   2. What are the impacts on your sleep quantity (how much you sleep)? What about sleep quality (how well you sleep)?
2. Does the night-time scratching (during the time you’d like to sleep) **of your [significant other]** have any impact on **your daily life**? [If yes, PROBES:]
   1. What are these impacts?
      1. [to elaborate] Can you tell me more about that, or give an example from a recent time?
3. Does the night-time scratching (during the time you’d like to sleep) **of your [significant other]** have any impact on **your work or school life**? [If yes, PROBES:]
   1. What are these impacts?
      1. [to elaborate] Can you tell me more about that, or give an example from a recent time?
4. Does night-time scratching (during the time you’d like to sleep) **of your [significant other]** have any impact on **your social life**? [If yes, PROBES:]
   1. What are these impacts?
      1. PROBE: Can you tell me more about that, or give an example from a recent time?

**Closing the interview:**

Thank you for your time today. The audio recording will stop after we close this session.

## **Appendix S3. Qualitative Interview Questions** for: Children with AD (aged 7–17 years)

**Introduction to the interview**

“Hello! My name is ____________ and I will now ask you about your eczema, and what your eczema makes you do when you are asleep. We will ask about itching and scratching, and by these we mean 2 things. When you itch it is the feeling on your skin. When you scratch, it is what you do when you rub your skin. It is just like when you are hungry, it is a feeling, and when you eat, it is what you do.

I will record what we talk about to help me think about it later. If you decide you don’t want to continue, we can stop at any time.”

**Understanding of meaningful aspects of daily life with eczema**

**Introduction:** Let’s start with some questions about your eczema.

1. Can you tell me about your eczema? What happens to you when you have eczema?
   1. PROBES:
      1. [to elaborate] Can you tell me more about that, or give me an example?
2. How do you know you have eczema? What kind of symptoms do you have?
   1. Which symptoms are the worst?
3. How do you feel about your eczema?
   1. PROBES:
      1. Is it ok, or does it bother you?
      2. PROBE: Can you tell me more about that, or give me an example?
4. Is there anything that you wish that you could do, but you can’t or don’t want to do because of your eczema?
   1. PROBES:
      1. Tell me more about the [activity] Would you do it more often if you didn’t have eczema?

**Focus on night-time scratching and expectations from treatment**:

Now we are going to ask some questions about scratching.

1. Do you sometimes scratch when you go to bed or while you are asleep?
   1. Do you scratch every night? How often?
   2. Is there any difference between when you scratch at night and when you scratch during the day? If yes, how is it different? Which is worse?
   3. Do you scratch when you lie down, before you fall asleep?
   4. Do you ever wake up because of the scratching? [If yes]
      1. How often? How long does it take you to go back to sleep? [Can use cues short/medium/long time to go back to sleep]
   5. Do you have to do anything to stop scratching at night?
   6. Do you sometimes scratch more and sometimes less at night?
   7. Does it sometimes leave any marks on your skin if you scratch at night?
2. Do you think if you didn’t scratch it would help your eczema get better?
   1. PROBES [if indicated scratching at night]:
      1. Would you like to scratch less when you go to sleep?
      2. Would you like to sleep better without the scratching?

**Understanding of broader impact of scratching on patient’s life**

1. If you scratch at night, what does the next day look like for you?
   1. Is there anything that you can’t or won’t do during the next day because you scratched at the night before?
2. [For older children] If you scratch at night, what does the next day in school look like for you?
   1. PROBES: Can you pay attention in school? Can you play sports? Play with friends? What about other activities?
3. [For older children] Would you wear some kind of thing, like a watch, to tell you more about your eczema and your symptoms?
   1. If yes, why? If no, why not?

**Closing the interview:**

Thank you for your time today. We are now finished, and when we say goodbye the recording will stop as well.

## **Appendix S4. Qualitative Interview Questions** for: Primary adult caregivers (>18 years of age) for children (aged 7–17) with atopic dermatitis

**Introduction to the interview**

“Welcome to this interview that is a part of the *Study to identify meaningful aspects of health connected to the symptom of nocturnal scratching in patients with atopic dermatitis.* My name is __________ and I will perform the interview with you today.

Please allow me to remind you that this interview is audio recorded. You can decide to withdraw at any time during our talk for any reason. In such case, just tell me if you wish not to continue.

In this interview we will focus on your and your child’s life and everyday experience with atopic dermatitis, often called eczema. We will also look more into your observations of their eczema and its symptoms. We will ask about their **night-time symptoms**. The night-time symptoms happen from when they get into bed until they wake up. Therefore, by saying “night-time symptoms”, we actually mean “symptoms during the time when you and your children intend to/would like to sleep, and the time when you do actually sleep, until you get out of bed”.

We will also use terms **itch** and **scratch**, and by these we don’t mean the same thing. We will refer to “itch” as the feeling, the sensation, the feeling of itchiness. We’ll use the term “scratch” as the action of scratching, meaning something one can do to their skin, the action. It is just like when you are hungry, it is a feeling, and when you eat, it is the action you do. It also doesn’t mean using only fingers or hand to do the motion of scratching. It can be any physical action or motion on the skin or clothing. It’s important to realize that itching and scratching can happen together, or they can happen on their own.”

**Understanding of meaningful aspects of daily life as a caregiver for a child with eczema**

**Introduction:** Let’s start with asking a few questions about your child’s eczema in general

1. Can you tell me about the eczema of your child since the time **he/she** has been diagnosed?
   1. PROBE: Can you tell me more about that, or give me an example from a recent time?
   2. When did it start? Is it getting better, or worse?
2. In your opinion, does your child’s eczema impact **his/her** daily life at all? [If yes, PROBES:]
   1. How?
   2. How does it impact their school life? Their home life? The activities they want to do?
   3. PROBE: Can you tell me more about that, or give me an example from a recent time?
3. What are the symptoms of eczema that you observe have the biggest impact on your child’s daily life?
   1. What symptoms do you feel are the most frustrating for your child?
   2. Which of them are the most bothersome for you?
4. Does your child’s eczema have any impact on **your** daily life? [If yes, probes:]
   1. What are these impacts?
   2. PROBE: Can you tell me more about that, or give an example from a recent time?
   3. What are the symptoms of eczema that you feel have the biggest impact on your daily life?

**Focus on nocturnal scratching and expectations from treatment**

**Introduction:** Now we want to just focus on the symptom of night-time scratching.

1. Have you observed that your child would scratch after they go to bed? [If yes, PROBES:]
   1. How did you observe this?
      1. Have you ever found evidence of scratching in the morning, (scratch marks, skin flakes, blood on sheets, etc.) even if you or child or you don’t remember scratching themselves?
      2. What was the evidence you observed? Can you elaborate?
      3. Have you witnessed any of the following: skin redness, skin thickness, flaking, crusting or oozing? If yes, which?
      4. How often does this happen?
   2. Does **your child’s** scratch during this time differ from scratching during the regular awake time? How?
      1. When do you think is it most bothersome?
   3. Does **your child** scratch after they go to bed, but before they fall asleep?
      1. If yes, how often does it happen?
   4. Have you noticed scratching would wake **your child** from sleep? [If yes] How often? How long does it take them to go back to sleep?
      1. Does it wake **you** from sleep? [If yes] How often?
   5. Do you do anything special to prevent or mitigate **your child’s** scratching during sleep time? Can you give some examples?
      1. Do you have any other specific night-time routines specific to your child’s eczema?
   6. Does **your child’s** night-time scratching vary across a longer period of time?
      1. If yes, are there nights when they scratch more, and nights when they scratch less?
      2. How does the scratching at night change when your child has a flare-up or your disease gets worse?
   7. Does the night-time scratching leave any marks on your child’s skin? [If yes] How often does it happen?
2. Do you think it is important that a treatment for eczema to lessen the overall scratching of your child? [If yes, PROBES:]
   1. Is it important to you that a treatment would also lessen their night-time scratching (during the sleep time)?
      1. What would you consider an improvement in your child’s night-time scratching?
         1. Probes: Would it be less intense / less often / last shorter of the scratching episodes - or other?
   2. Do you think reducing your child’s night-time scratching would improve **their everyday life**? [If yes, PROBES:]
      1. How?
   3. Do you think reducing your child’s night-time scratching would improve **their sleep**? [If yes, PROBES:]
      1. How?
      2. PROBES: fall asleep faster, wake less often from their sleep - or other?
   4. Do you think it would bring any improvements for **you** if your child has better nights less disturbed by their night-time scratching? [If yes, PROBES:]
      1. What would be those improvements?
3. We understand it may be hard to tell the amount of scratching at night, or if it is getting better or worse. If there was a way to tell you about the amount of your child’s scratching at night, would this be valuable to you?
   1. How would you use this information?
   2. Would you be willing to permit your child to wear a tool or device to capture this data?
      1. If yes, why? If no, why not?
   3. Would you be willing to permit use of some kind of “room sensor” which could do this, but would not be worn directly?
      1. If yes, why? If no, why not?

**Understanding of broader impact of scratching on the child’s life**

**Introduction:** Now we want to focus on what are some broader impacts of your child’s night-time scratching on your child’s life.

1. Does the night-time scratching (during the time you’d like to sleep) **of your child** have any impact on **their sleep or the quality of their sleep**? [If yes, PROBES:]
   1. What are these impacts?
      1. PROBE: Can you tell me more about that, or give an example from a recent time?
2. Does the night-time scratching (during the time you’d like to sleep) **of your child** have any impact on **their daily life**? [If yes, probes:]
   1. What are these impacts?
      1. PROBE: Can you tell me more about that, or give an example from a recent time?
3. Does the night-time scratching (during the time you’d like to sleep) **of your child** have any impact on **their work or school life**? [If yes, PROBES:]
   1. What are these impacts?
      1. [to elaborate] Can you tell me more about that, or give an example from a recent time?
4. Does night-time scratching (during the time you’d like to sleep) **of your child** have any impact on **their social life**? [If yes, PROBES:]
   1. What are these impacts?
      1. PROBE: Can you tell me more about that, or give an example from a recent time?

**Understanding of broader impact of scratching on caregiver’s life**

**Introduction:** Now we want to focus on what are some broader impacts of your child’s night-time scratching on your life as a parent or caregiver.

1. Does the night-time scratching (during the time you’d like to sleep) **of your child** have any impact on **your sleep or the quality of your sleep**? [If yes, PROBES:]
   1. What are these impacts?
      1. PROBE: Can you tell me more about that, or give an example from a recent time?
2. Does the night-time scratching (during the time you’d like to sleep) **of your child** have any impact on **your work or school life**? [If yes, PROBES:]
   1. What are these impacts?
      1. PROBE: Can you tell me more about that, or give an example from a recent time?
3. Does night-time scratching (during the time you’d like to sleep) **of your child** have any impact on **your social life**? [If yes, PROBES:]
   1. What are these impacts?
      1. PROBES:
         1. Can you tell me more about that, or give an example from a recent time?
         2. How about social gatherings? Vacations? etc.

**Closing the interview:**

Thank you for your time today. The audio recording will stop after we close this session.

## **Appendix S5. Cognitive Testing Interview,** Adult patient

| Introduction | Ask respondent to read the instructions to themselves. |
| --- | --- |
|  | In this survey, we will focus on your life and everyday experience with atopic dermatitis, which we will refer to here as eczema. We ask about your eczema, its symptoms, and specifically your night-time symptoms. Night-time symptoms include those that you experience from the time you go to bed to sleep, during the time when you do actually sleep, and until you get out of bed after you wake up.  We will also use the terms itch and scratch. Often people use these to mean the same thing, but we will use them to mean different things in this survey. We will refer to “itch” as the sensation that you have, the feeling of itchiness you perceive. We’ll use the term “scratch” as the action of scratching, meaning something you can do to your skin. Scratching also doesn’t necessarily mean you are using your fingers to do the scratching. It can be any physical action or motion on the skin or clothing.  Another way to think about the difference between itch and scratch is just like when you are hungry. Hunger is a feeling like itching. Eating, which is an action, is like scratching. It’s important to realize that they can happen together, or they can happen on their own. For example, you feel itchy, so you start scratching. Or, you can feel itchy and not scratch. Or, you can scratch yourself without feeling itchy.  Probe/check for: |
|  |  |
| Comprehension | - Difficulty understanding or misreading the instructions - Did not fully read instructions - Clarify respondent’s understanding of itch and scratch |
|  |  |
| Section 1 | Ask respondent to read the instructions to themselves |
|  | In this section we’ll ask you a few questions about your eczema and symptoms in general. Remember that you feel itchy, and scratching is the action.  When we ask about something happening during the day, we mean from the time you are out of bed after waking up, until the time you go to bed to sleep. When we ask about something happening at night, we mean from the time you go to bed to sleep, to the time you wake up and get out of bed. |
| Comprehension | Probe/check for: |
|  | - Difficulty understanding or misreading the instructions - Did not fully read instructions - Clarify respondent’s understanding of itch and scratch |
| Question 1 | Did a doctor tell you that you have eczema? |
|  | Probe/check for: |
|  |  |
| Comprehension | - Difficulty understanding or misreading the question   Not fully reading the instructions |
|  |  |
| Retrieval | - Had Insufficient knowledge to answer - Focused on only one part of the question |
|  |  |
| Response | - Response made too quickly - Not reading all options - Provided revised option choice during interview. |
|  |  |
| Judgment | - Felt uncomfortable or reluctant providing an answer. e.g., stigma. |
| Question 2 | How would you describe the severity of your eczema over the past 2 weeks? |
| Comprehension | - Difficulty understanding or misreading the question   Not fully reading the instructions |
|  |  |
| Retrieval | - Had Insufficient knowledge to answer - Focused on only one part of the question |
|  |  |
| Response | - Response made too quickly - Not reading all options - Provided revised option choice during interview. |
|  |  |
| Judgment | - Felt uncomfortable or reluctant providing an answer. e.g., stigma. |
| Question 3 | During the past 2 weeks, how often did you experience the following? |
|  |  |
| Comprehension | - What is the respondent’s interpretation of ‘past 2 weeks’ |
|  | - Not fully reading the question - Clarify respondent’s understanding of itch and scratch |
|  |  |
| Retrieval | - Insufficient knowledge to answer |
|  | - Focused on only one part of the question |
|  |  |
| Judgment | - Felt uncomfortable or reluctant providing an answer. e.g., stigma. |
|  |  |
| Response (Symptoms) | - Symptoms missing - Not reading all options |
|  |  |
| Response  (Frequency selection) | - Adequacy of options - Not reading all options - Suggestions for change |
|  |  |
| Question 4 | During the past 2 weeks, how intense were the following? |
|  |  |
| Comprehension | - Difficulty understanding or misreading the question   Not fully reading the instructions |
|  |  |
| Retrieval | - Had Insufficient knowledge to answer - focused on only one part of the question |
|  |  |
| Response | - Response made too quickly - Not reading all options - Provided revised option choice during interview. |
|  |  |
| Judgment | - Felt uncomfortable or reluctant providing an answer. e.g., stigma. |
| Question 5 | During the past 2 weeks, how burdensome did you find the following? |
|  | Probe/check for: |
|  |  |
| Comprehension | - Respondent’s understanding of ‘burdensome’ |
|  | - Not fully reading the question - Clarify respondent’s understanding of itch and scratch |
|  |  |
| Retrieval | - Insufficient knowledge to answer |
|  |  |
| Judgment | - Felt uncomfortable or reluctant providing an answer. e.g., stigma. |
|  |  |
| Response  (Options) | - Symptoms missing - Not reading all options |
|  |  |
| Responses  (Frequency) | - Adequacy of options |
|  | - Suggestions for change |
|  |  |
| Question 6 | Rank the following from most to least burdensome. Please start with the most burdensome at the top. |
|  | Probe/check for: |
|  |  |
| Comprehension | - Respondent’s understanding of ‘rank’ - Clarify respondent’s understanding of itch and scratch |
|  |  |
| Question 7 | To what extent does your eczema limit the following aspects of your life? |
|  | Probe/check for: |
|  |  |
| Comprehension | - Difficulty understanding or misreading the question |
|  |  |
| Retrieval | - Insufficient knowledge to answer |
|  |  |
| Response | - Not reading all options |
|  | - Adequacy of options |
|  | - Suggestions for change |
|  |  |
|  | Ask respondent to read the instructions to themselves |
| Section 2: | In this section we’ll ask you a few questions about your eczema and symptoms in general. Remember that you feel itchy, and scratching is the action.  When we ask about something happening during the day, we mean from the time you are out of bed after waking up, until the time you go to bed to sleep. When we ask about something happening at night, we mean from the time you go to bed to sleep, to the time you wake up and get out of bed. |
| Comprehension | Probe/check for: |
|  |  |
|  | - Difficulty understanding or misreading the instructions - Did not fully read instructions - Clarify respondent’s understanding of itch and scratch |
| Question 8 | During the past 2 weeks, how often did you scratch during the day (during the time when you would like to be awake) because of your eczema?  Probe/check for: |
|  |  |
| Comprehension | - Difficulty understanding or misreading the instructions - Did not fully read instructions - Clarify respondent’s understanding of itch and scratch |
|  |  |
| Retrieval | - Insufficient knowledge to answer |
|  |  |
| Response | - Not reading all options |
|  | - Adequacy of options |
|  |  |
| Question 9 | How aware are you of your night-time scratching? |
| Comprehension | - Difficulty understanding or misreading the question   Not fully reading the instructions |
|  |  |
| Retrieval | - Had Insufficient knowledge to answer - Focused on only one part of the question |
|  |  |
| Response | - Response made too quickly - Not reading all options - Provided revised option choice during interview. |
|  |  |
| Judgment | - Felt uncomfortable or reluctant providing an answer. e.g., stigma. |
| Question 10 | During the past 2 weeks, how often did you scratch at night (during the time when you would like to sleep) because of your eczema? |
|  | Probe/check for: |
|  |  |
| Comprehension | - Difficulty understanding or misreading the question - Clarify respondent’s understanding of itch and scratch |
| Retrieval | - Insufficient knowledge to answer |
|  |  |
| Response | - Not reading all options |
|  | - Adequacy of options |
|  | - Suggestions for change |
| Question 11 | During the past 2 weeks, how often did you wake up with the following signs that weren’t there when you went to bed? |
|  | Probe/check for: |
|  |  |
| Comprehension | - Difficulty understanding or misreading the question - Clarify respondent’s understanding of itch and scratch |
|  |  |
| Retrieval | - Insufficient knowledge to answer |
|  |  |
|  | - Not reading all options |
|  |  |
| Response | - Not reading all options |
|  | - Adequacy of options |
|  | - Suggestions for change |
| Question 12 | During the past 2 weeks, how often did your night-time scratching affect your sleep? |
|  |  |
| Comprehension | - Difficulty understanding or misreading the question - Clarify respondent’s understanding of itch and scratch |
|  |  |
| Retrieval | - Insufficient knowledge to answer |
|  | - Not reading all options |
|  |  |
| Response | - Not reading all options |
|  | - Adequacy of options |
|  | - Suggestions for change |
| Question 13 | During the past 2 weeks, on average, how many hours of sleep did you lose because of night-time scratching? |
|  | Probe/check for: |
|  |  |
| Comprehension | - Difficulty understanding or misreading the question(e.g., mitigate) - Clarify respondent’s understanding of itch and scratch |
|  |  |
| Retrieval | - Insufficient knowledge to answer |
|  |  |
|  | - Not reading all options |
|  |  |
| Response | - Not reading all options |
|  | - Adequacy of options |
|  | - Suggestions for change |
|  |  |
| Question 14 | During the past 2 weeks, how often has your night-time scratching (during the time when you would like to sleep) impacted each of the following aspects of your life? |
|  | Probe/check for: |
|  |  |
| Comprehension | - Difficulty understanding or misreading the question - Clarify respondent’s understanding of itch and scratch |
|  |  |
| Retrieval | - Insufficient knowledge to answer |
|  |  |
|  | - Not reading all options |
|  |  |
| Response | - Not reading all options |
|  | - Adequacy of options |
|  | - Suggestions for change |

|  |  |
| --- | --- |
| Question 15 | Do you do any of the following to prevent or reduce your night-time scratching (during the time when you would like to sleep)? |
|  | Probe/check for: |
|  |  |
| Comprehension | - Difficulty understanding or misreading the question - Clarify respondent’s understanding of itch and scratch |
|  |  |
| Retrieval | - Insufficient knowledge to answer |
|  |  |
|  | - Not reading all options |
|  |  |
| Response | - Not reading all options |
|  | - Adequacy of options |
|  | - Suggestions for change |

| Question 16 | How important is it that an eczema treatment lessens your night-time scratching (during the time when you would like to sleep)? |
| --- | --- |
|  | Probe/check for: |
|  |  |
| Comprehension | - Difficulty understanding or misreading the question - Clarify respondent’s understanding of itch and scratch |
|  |  |
| Retrieval | - Insufficient knowledge to answer |
|  |  |
|  | - Not reading all options |
|  |  |
| Response | - Not reading all options |
|  | - Adequacy of options |
|  | - Suggestions for change |

| Question 17 | What improvements connected to your night-time scratching (during the time when you would like to sleep) are most important to you? |
| --- | --- |
|  | Probe/check for: |
|  |  |
| Comprehension | - Difficulty understanding or misreading the question - Clarify respondent’s understanding of itch and scratch |
|  |  |
| Retrieval | - Insufficient knowledge to answer |
|  |  |
|  | - Not reading all options |
|  |  |
| Response | - Not reading all options |
|  | - Adequacy of options |
|  | - Suggestions for change |

| Question 18 | How valuable would it be to measure your night-time scratching (during the time when you would like to sleep)? |
| --- | --- |
|  | Probe/check for: |
|  |  |
| Comprehension | - Difficulty understanding or misreading the question - Clarify respondent’s understanding of itch and scratch |
|  |  |
| Retrieval | - Insufficient knowledge to answer |
|  |  |
|  | - Not reading all options |
|  |  |
| Response | - Not reading all options |
|  | - Adequacy of options |
|  | - Suggestions for change |

| Question 19 | Would you be willing to use technology (for example, a wearable device or bed-mounted device) to measure your night-time scratching? |
| --- | --- |
|  | Probe/check for: |
|  |  |
| Comprehension | - Difficulty understanding or misreading the question - Clarify respondent’s understanding of itch and scratch |
|  |  |
| Retrieval | - Insufficient knowledge to answer |
|  |  |
|  | - Not reading all options |
|  |  |
| Response | - Not reading all options |
|  | - Adequacy of options |
|  | - Suggestions for change |

| Question 20 | How would you use the information collected about your night-time scratching by this technology? |
| --- | --- |
|  | Probe/check for: |
|  |  |
| Comprehension | - Difficulty understanding or misreading the question - Clarify respondent’s understanding of itch and scratch |
|  |  |
| Retrieval | - Insufficient knowledge to answer |
|  |  |
|  | - Not reading all options |
|  |  |
| Response | - Not reading all options |
|  | - Adequacy of options |
|  | - Suggestions for change |
| Question 21 | Which of the following concerns you about using technology to measure your night-time scratching (during the time when you would like to sleep)? |
|  |  |
| Comprehension | - Difficulty understanding or misreading the question   Not fully reading the instructions |
|  |  |
| Retrieval | - Had Insufficient knowledge to answer - Focused on only one part of the question |
|  |  |
| Response | - Response made too quickly - Not reading all options - Provided revised option choice during interview. |
|  |  |
| Judgment | - Felt uncomfortable or reluctant providing an answer. e.g., stigma. |

## **Appendix S6. Cognitive Testing Interview,** Caregiver for child patient

| Introduction | Ask respondent to read the instructions to themselves. |
| --- | --- |
|  | Probe/check for: |
|  | In this survey, we will focus on the life and everyday experiences of your child with atopic dermatitis, which we will refer to here as eczema. We ask about your child’s eczema, their symptoms, and specifically their night-time symptoms. Night-time symptoms include those that your child may experience from the time they go to bed to sleep, during the time when they do actually sleep, and until they get out of bed after they wake up.  We will also use the terms itch and scratch, and by these we don’t mean the same thing. We will refer to “itch” as the sensation that your child has, the feeling of itchiness they perceive. We’ll use the term “scratch” as the action of scratching, meaning something your child can do to their skin. Scratching also doesn’t necessarily mean your child is using their fingers to do the scratching. It can be any physical action or motion on the skin or clothing.  Another way to think about the difference between itch and scratch is just like when you are hungry. Hunger is a feeling like itching. Eating, which is an action, is like scratching. It’s important to realize that they can happen together, or they can happen on their own. For example, you feel itchy, so you start scratching. Or, you can feel itchy and not scratch. Or, you can scratch yourself without feeling itchy. |
| Comprehension | - Difficulty understanding or misreading the instructions - Did not fully read instructions - Clarify respondent’s understanding of itch and scratch |
| Section 1 | Ask respondent to read the instructions to themselves |
|  | In this section we’ll ask you a few questions about your child’s eczema and symptoms in general. Remember that you feel itchy, and scratching is the action.  When we ask about something happening during the day, we mean from the time you are out of bed after waking up, until the time you go to bed to sleep. When we ask about something happening at night, we mean from the time you go to bed to sleep, to the time you wake up and get out of bed. |
| Comprehension | Probe/check for: |
|  |  |
|  | - Difficulty understanding or misreading the instructions - Did not fully read instructions - Clarify respondent’s understanding of itch and scratch |
| Question 1 | Did a doctor tell you and/or your child that your child has eczema? |
|  | Probe/check for: |
|  |  |
| Comprehension | - Difficulty understanding or misreading the question - Not fully reading the instructions |
|  |  |
| Retrieval | - Had Insufficient knowledge to answer - Focused on only one part of the question |
|  |  |
| Response | - Response made too quickly - Not reading all options - Provided revised option choice during interview. |
|  |  |
| Judgment | - Felt uncomfortable or reluctant providing an answer. e.g., stigma. |
| Question 2 | How would you describe the severity of your child’s eczema over the past 2 weeks? |
| Question 3 | During the past 2 weeks, how often did you observe the following on your child? |
|  | Probe/check for: |
|  |  |
| Comprehension | - What is the respondent’s interpretation of ‘past 2 weeks’ |
|  | - Not fully reading the question - Clarify respondent’s understanding of itch and scratch |
|  |  |
| Retrieval | - Insufficient knowledge to answer |
|  | - Focused on only one part of the question |
|  |  |
| Judgment | - Felt uncomfortable or reluctant providing an answer. e.g., stigma. |
|  |  |
| Response (Symptoms) | - Symptoms missing - Not reading all options |
|  |  |
| Response  (Frequency selection) | - Adequacy of options - Not reading all options - Suggestions for change |
|  |  |
| Question 4 | During the past 2 weeks, what is your perception of how intense your child found the following? |
| Question 5 | During the past 2 weeks, what is your perception of how burdensome your child found the following? |
|  | Probe/check for: |
|  |  |
| Comprehension | - Respondents understanding of ‘burdensome’ |
|  | - Not fully reading the question - Clarify respondent’s understanding of itch and scratch |
|  |  |
| Retrieval | - Insufficient knowledge to answer |
|  |  |
| Judgment | - Felt uncomfortable or reluctant providing an answer. e.g., stigma. |
|  |  |
| Response  (Options) | - Symptoms missing - Not reading all options |
|  |  |
| Responses  (Frequency) | - Adequacy of options |
|  | - Suggestions for change |
| Question 6 | Rank the following from most to least burdensome for your child. Please start with the most burdensome symptom at the top. |
|  | Probe/check for: |
|  |  |
| Comprehension | - Respondent’s understanding of ‘rank’ - Clarify respondent’s understanding of itch and scratch |
| Question 7 | To what extent does your child’s eczema limit the following aspects of their life? |
|  | Probe/check for: |
|  |  |
| Comprehension | - Difficulty understanding or misreading the question |
|  |  |
| Retrieval | - Insufficient knowledge to answer |
|  |  |
| Response | - Not reading all options |
|  | - Adequacy of options |
|  | - Suggestions for change |
|  |  |
|  |  |
| Section 2 | Ask respondent to read the instructions to themselves. |
|  | Probe/check for: |
|  | In this section we’ll ask you a few questions about your child’s eczema and symptoms in general. Remember that your child feels itchy, and scratching is the action.  When we ask about something happening during the day, we mean from the time your child is out of bed after waking up, until the time they go to bed to sleep. When we ask about something happening at night, we mean from the time your child goes to bed to sleep, to the time they wake up and get out of bed. |
| Comprehension | - Difficulty understanding or misreading the instructions - Did not fully read instructions - Clarify respondent’s understanding of itch and scratch |
| Question 8 | During the past 2 weeks, how often did you observe your child scratching during the day (during the time when they would like to be awake)? |
|  | Probe/check for: |
|  |  |
| Comprehension | - Difficulty understanding or misreading the instructions - Did not fully read instructions - Clarify respondent’s understanding of itch and scratch |
|  |  |
| Retrieval | - Insufficient knowledge to answer |
|  |  |
| Response | - Not reading all options |
|  | - Adequacy of options |
| Question 9 | How aware are you of your child’s night-time scratching? |
|  |  |
| Comprehension | - Difficulty understanding or misreading the question |
|  |  |
| Retrieval | - Insufficient knowledge to answer |
|  |  |
| Response | - Not reading all options |
|  | - Adequacy of options |
|  | - Suggestions for change |
| Question 10 | During the past 2 weeks, how often did you observe your child scratching at night (during the time when they would like to sleep)? |
|  | Probe/check for: |
|  |  |
| Comprehension | - Difficulty understanding or misreading the question - Clarify respondent’s understanding of itch and scratch |
| Retrieval | - Insufficient knowledge to answer |
|  |  |
| Response | - Not reading all options |
|  | - Adequacy of options |
|  | - Suggestions for change |
| Question 11 | During the past 2 weeks, how often did your child wake up with the following signs that weren’t there when they went to bed? |
|  | Probe/check for: |
|  |  |
| Comprehension | - Difficulty understanding or misreading the question - Clarify respondent’s understanding of itch and scratch |
|  |  |
| Retrieval | - Insufficient knowledge to answer |
|  |  |
|  | - Not reading all options |
|  |  |
| Response | - Not reading all options |
|  | - Adequacy of options |
|  | - Suggestions for change |
| Question 12 | During the past 2 weeks, how often did your child’s night-time scratching affect your child’s sleep? |
|  |  |
| Comprehension | - Difficulty understanding or misreading the question - Clarify respondent’s understanding of itch and scratch |
|  |  |
| Retrieval | - Insufficient knowledge to answer |
|  | - Not reading all options |
|  |  |
| Response | - Not reading all options |
|  | - Adequacy of options |
| Question 13 | During the past 2 weeks, on average, how many hours of sleep did your child lose because of their night-time scratching? |
|  | Probe/check for: |
|  |  |
| Comprehension  Retrieval  Response | \| - Difficulty understanding or misreading the question - Clarify respondent’s understanding of itch and scratch \| \| --- \| \|  \| \| - Insufficient knowledge to answer \| \| - Not reading all options \| \|  \| \| - Not reading all options \| \| - Adequacy of options \| |
|  |  |
| Question 14 | During the past 2 weeks, how often has your child’s night-time scratching (during the time when they would like to sleep) impacted each of the following aspects of your child’s life? |
|  | Probe/check for: |
|  |  |
| Comprehension | - Difficulty understanding or misreading the question(e.g., mitigate) - Clarify respondent’s understanding of itch and scratch |
|  |  |
| Retrieval | - Insufficient knowledge to answer |
|  |  |
|  | - Not reading all options |
|  |  |
| Response | - Not reading all options |
|  | - Adequacy of options |
|  | - Suggestions for change |
| Question 15 | Does your child do any of the following to prevent or reduce their night-time scratching (during the time when they would like to sleep)? |
|  | Probe/check for: |
|  |  |
| Comprehension | - Difficulty understanding or misreading the question - Clarify respondent’s understanding of itch and scratch |
|  |  |
| Retrieval | - Insufficient knowledge to answer |
|  |  |
|  | - Not reading all options |
|  |  |
| Response | - Not reading all options |
|  | - Adequacy of options |
|  | - Suggestions for change |
| Question 16 | During the past 2 weeks, how burdensome did you find the following? |
| Comprehension | - Difficulty understanding or misreading the question - Respondents understanding of ‘burdensome’ |
|  |  |
| Retrieval | - Insufficient knowledge to answer |
|  |  |
| Response | - Not reading all options |
|  | - Adequacy of options |
|  | - Suggestions for change |
|  |  |
| Question 17 | Rank the following from most to least burdensome for you about your child’s eczema. Please start with the most burdensome symptom at the top. |
| Comprehension | - Difficulty understanding or misreading the question - Respondents understanding of ‘burdensome’ |
|  |  |
| Retrieval | - Insufficient knowledge to answer |
|  |  |
| Response | - Not reading all options |
|  | - Adequacy of options |
|  | - Suggestions for change |

| Question 18 | During the past 2 weeks, how often did your child’s night-time scratching affect your sleep? |
| --- | --- |
|  | Probe/check for: |
|  |  |
| Comprehension | - Difficulty understanding or misreading the question - Clarify respondent’s understanding of itch and scratch |
|  |  |
| Retrieval | - Insufficient knowledge to answer |
|  |  |
|  | - Not reading all options |
|  |  |
| Response | - Not reading all options |
|  | - Adequacy of options |
|  | - Suggestions for change |

| Question 19 | During the past 2 weeks, on average, how many hours of sleep did you lose because of your child’s night-time scratching? |
| --- | --- |
|  | Probe/check for: |
|  |  |
| Comprehension | - Difficulty understanding or misreading the question - Clarify respondent’s understanding of itch and scratch |
|  |  |
| Retrieval | - Insufficient knowledge to answer |
|  |  |
|  | - Not reading all options |
|  |  |
| Response | - Not reading all options |
|  | - Adequacy of options |
|  | - Suggestions for change |

| Question 20 | During the past 2 weeks, how often has your child’s night-time scratching (during the time when they would like to sleep) impacted each of the following aspects of your life? |
| --- | --- |
|  | Probe/check for: |
|  |  |
| Comprehension | - Difficulty understanding or misreading the question - Clarify respondent’s understanding of itch and scratch |
|  |  |
| Retrieval | - Insufficient knowledge to answer |
|  |  |
|  | - Not reading all options |
|  |  |
| Response | - Not reading all options |
|  | - Adequacy of options |
|  | - Suggestions for change |

| Question 21 | How important is it that an eczema treatment lessens your child’s night-time scratching (during the time when they would like to sleep)? |
| --- | --- |
|  | Probe/check for: |
|  |  |
| Comprehension | - Difficulty understanding or misreading the question - Clarify respondent’s understanding of itch and scratch |
|  |  |
| Retrieval | - Insufficient knowledge to answer |
|  |  |
|  | - Not reading all options |
|  |  |
| Response | - Not reading all options |
|  | - Adequacy of options |
|  | - Suggestions for change |

| Question 22 | What improvements connected to your child’s night-time scratching (during the time when they would like to sleep) are most important to you?  Probe/check for: |
| --- | --- |
|  |  |
| Comprehension | - Difficulty understanding or misreading the question - Clarify respondent’s understanding of itch and scratch |
|  |  |
| Retrieval | - Insufficient knowledge to answer |
|  |  |
|  | - Not reading all options |
|  |  |
| Response | - Not reading all options |
|  | - Adequacy of options |
|  | - Suggestions for change |
|  |  |

| Question 23 | How valuable would it be to measure your child’s night-time scratching (during the time when they would like to sleep)? |
| --- | --- |
|  | Check probe for: |
|  |  |
| Comprehension | - Difficulty understanding or misreading the question - Clarify respondent’s understanding of itch and scratch |
|  |  |
| Retrieval | - Insufficient knowledge to answer |
|  |  |
|  | - Not reading all options |
|  |  |
| Response | - Not reading all options |
|  | - Adequacy of options |
|  | - Suggestions for change |
|  |  |

| Question 24 | Would you be willing to have your child use technology (for example, a wearable device or bed-mounted device) to measure their night-time scratching? |
| --- | --- |
|  | Check probe for: |
|  |  |
| Comprehension | - Difficulty understanding or misreading the question - Clarify respondent’s understanding of itch and scratch |
|  |  |
| Retrieval | - Insufficient knowledge to answer |
|  |  |
|  | - Not reading all options |
|  |  |
| Response | - Not reading all options |
|  | - Adequacy of options |
|  | - Suggestions for change |
|  |  |
| Question 25 | How would you use the information collected about your child’s night-time scratching by this technology? |
| Comprehension | - Difficulty understanding or misreading the question - Clarify respondent’s understanding of itch and scratch |
|  |  |
| Retrieval | - Insufficient knowledge to answer |
|  |  |
|  | - Not reading all options |
|  |  |
| Response | - Not reading all options |
|  | - Adequacy of options |
|  | - Suggestions for change |
| Question 26 | Which of the following concerns you about using technology to measure your child’s night-time scratching (during the time when they would like to sleep)? |
| Comprehension | - Difficulty understanding or misreading the question   Not fully reading the instructions |
|  |  |
| Retrieval | - Had Insufficient knowledge to answer - focused on only one part of the question |
|  |  |
| Response | - Response made too quickly - Not reading all options - Provided revised option choice during interview. |
|  |  |
| Judgment | - Felt uncomfortable or reluctant providing an answer. e.g., stigma. |

## Appendix S7. Quantitative survey: Adult patient

**Introduction**

In this survey, we will focus on your life and everyday experience with atopic dermatitis, which we will refer to here as eczema. We ask about your eczema, its symptoms, and specifically your night-time symptoms. Night-time symptoms include those that you experience from the time you go to bed to sleep, during the time when you do actually sleep, and until you get out of bed after you wake up.

We will also use the terms itch and scratch. Often people use these to mean the same thing, but we will use them to mean different things in this survey. We will refer to “itch” as the sensation that you have, the feeling of itchiness you perceive. We’ll use the term “scratch” as the action of scratching, meaning something you can do to your skin. Scratching also doesn’t necessarily mean you are using your fingers to do the scratching. It can be any physical action or motion on the skin or clothing.

Another way to think about the difference between itch and scratch is just like when you are hungry. Hunger is a feeling like itching. Eating, which is an action, is like scratching. It’s important to realize that they can happen together, or they can happen on their own. For example, you feel itchy, so you start scratching. Or, you can feel itchy and not scratch. Or, you can scratch yourself without feeling itchy.

Section 1:

In this section we’ll ask you a few questions about your eczema and symptoms in general. Remember that you feel itchy, and scratching is the action.

When we ask about something happening during the day, we mean from the time you are out of bed after waking up, until the time you go to bed to sleep. When we ask about something happening at night, we mean from the time you go to bed to sleep, to the time you wake up and get out of bed.

1. Did a doctor tell you that you have eczema?
   - Yes
   - No
2. Based on the introduction above, which one of the following options describes ITCH?
   - The sensation, feeling on your skin
   - The action, something you do to your skin
3. How would you describe the severity of your eczema:
   - Over the past 2 weeks
   - When it’s at its worst

Scale:

- - None
  - Mild
  - Moderate
  - Severe
  - Very severe

1. During the past 2 weeks, how often did you experience the following?

- Itchy skin because of your eczema (remember: itchy is the feeling you get)
- Issues with sleep because of your eczema
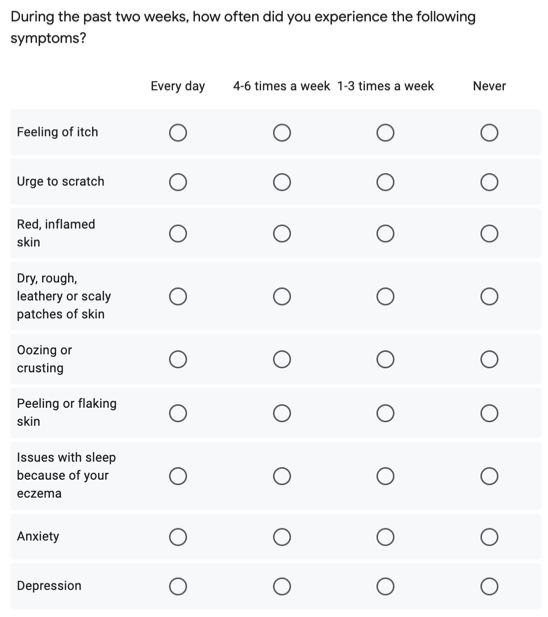

- Feelings of anxiety
- Feelings of depression
- Day-time scratching
- Night-time scratching
- Red, inflamed skin
- Dry, rough, leathery or scaly patches of skin
- Oozing or crusting
- Peeling or flaking skin

Frequency selection:

- Multiple times a day
- Every day
- 5-6 days a week
- 3-4 days a week
- 1-2 days a week
- No days
- Don’t know/unsure

1. During the past 2 weeks, how intense were the following?
   - Day-time itchy skin because of your eczema
   - Day-time scratching
   - Night-time itchy skin because of your eczema
   - Night-time scratching

Intensity scale:

- Not at all
- Slightly
- Moderately
- Quite a bit
- Extremely

1. During the past 2 weeks, how burdensome did you find the following?

- Itchy skin because of your eczema
- Issues with sleep because of your eczema
- Feelings of anxiety
- Feelings of depression
- Day-time scratching
- Night-time scratching
- Red, inflamed skin
- Dry, rough, leathery or scaly patches of skin
- Oozing or crusting
- Peeling or flaking skin

Intensity selection:

- Scale with anchors for each point
  - Not at all
  - Slightly
  - Moderately
  - Quite a bit
  - Extremely

1. Rank the following from most to least burdensome. Please start with the most burdensome at the top.
   - Itchy skin
   - Day-time scratching
   - Night-time scratching
   - Red, inflamed skin
   - Dry, rough, leathery or scaly patches of skin
   - Oozing or crusting
   - Peeling or flaking skin
   - Issues with sleep because of your eczema
   - Feelings of anxiety
   - Feelings of depression
2. To what extent does your eczema limit the following aspects of your life? [5-pt scale]
   - Being spontaneous
   - Playing a sport or exercising
   - Selecting clothes
   - Eating
   - Choosing a job or career
   - Attending social or public events
   - Forming relationships with peers
   - Forming romantic relationships
   - Other [with freeform input]

- Scale
  - Not at all
  - Slightly
  - Moderately
  - Quite a bit
  - Extremely

Section 2:

In this section we’ll ask you a few questions about your eczema and symptoms in general. Remember that you feel itchy, and scratching is the action.

When we ask about something happening during the day, we mean from the time you are out of bed after waking up, until the time you go to bed to sleep. When we ask about something happening at night, we mean from the time you go to bed to sleep, to the time you wake up and get out of bed.

1. During the past 2 weeks, how often did you scratch during the day (during the time when you would like to be awake) because of your eczema? [checkboxes]
   - Multiple times a day
   - Every day
   - 5-6 days a week
   - 3-4 days a week
   - 1-2 days a week
   - No days
   - Don’t know/unsure
2. How aware are you of your night-time scratching?

- Not at all
- Slightly
- Moderately
- Quite a bit
- Extremely

1. During the past 2 weeks, how often did you scratch at night (during the time when you would like to sleep) because of your eczema? [checkboxes]
   - Multiple times a night
   - Every night
   - 5-6 nights a week
   - 3-4 nights a week
   - 1-2 nights a week
   - No nights
   - Don’t know/unsure
2. During the past 2 weeks, how often did you wake up with the following signs that weren’t there when you went to bed?
   - Skin flakes
   - Skin cuts
   - Blood on sheets or skin
   - Open wounds or torn skin
   - Scratches on skin/scratch marks
   - Crusting
   - Oozing
   - Irritated or red skin
   - Thickening of skin

Frequency

- - Multiple times a night
  - Every night
  - 5-6 nights a week
  - 3-4 nights a week
  - 1-2 nights a week
  - No nights
  - Don’t know/unsure

1. During the past 2 weeks, how often did your night-time scratching affect your sleep?
   - Every night
   - 5-6 nights a week
   - 3-4 nights a week
   - 1-2 nights a week
   - No nights
   - Don’t know/unsure
2. During the past 2 weeks, on average, how many hours of sleep did you lose because of night-time scratching? [0-12 hour sliding scale]
3. During the past 2 weeks, how often has your night-time scratching (during the time when you would like to sleep) impacted each of the following aspects of your life?
   - Not sleeping at all at night
   - Not feeling rested in the morning, feeling tired during the day
   - Not being able to focus during the day
   - Lowered productivity at work or school
   - Missing work or school completely
   - Missing out on social life and activities with your family, friends, neighbors, etc.
   - Not being able to take vacations or travel
   - Feeling depressed or anxious during the day
   - Less overall happiness
   - Seeking a therapy or a specialist to help with sleep issues
   - Seeking medical attention for skin issues
   - Impacted intimacy in relationships
   - None of the above
   - Other [with freeform input]

Frequency selection:

- Every day
- 5-6 days a week
- 3-4 days a week
- 1-2 days a week
- No days
- Don’t know/unsure

1. Do you do any of the following to prevent or reduce your night-time scratching (during the time when you would like to sleep)? [checkboxes]
   - Wear special clothes (e.g., long-sleeved or made of specific material)
   - Wrap hands or use gloves for the time you sleep
   - Shower before going to sleep
   - Apply creams, lotions or topical medications before sleep
   - Take bleach baths or use wet wraps before sleep
   - Take oral eczema medications before sleep
   - Take over-the-counter sleep medication(s)
   - Take prescription sleep medication(s)
   - Practice meditation or focused breathing
   - Lower the temperature in the room where you sleep
   - Go to the doctor to change my treatment regimen
   - None of the above
   - Other [with freeform input]
2. How important is it that an eczema treatment lessens your night-time scratching (during the time when you would like to sleep)?
   - Not important at all
   - Slightly important
   - Moderately important
   - Quite a bit important
   - Extremely important
3. What improvements connected to your night-time scratching (during the time when you would like to sleep) are most important to you? [checkboxes]
   - Spending less total time scratching during the night
   - Having shorter scratching episodes during the night
   - Having fewer scratching episodes during the night
   - Reducing how hard I scratch at night
   - Waking less often during the night due to scratching
   - Having less new skin damage when I wake up
   - Having better sleep quality in general
   - Being less itchy during the night
   - Falling asleep more easily
   - None of the above
   - Other [with freeform input]
4. How valuable would it be to measure your night-time scratching (during the time when you would like to sleep)?
   - Not at all
   - Slightly
   - Moderately
   - Quite a bit
   - Extremely
5. Would you be willing to use technology (for example, a wearable device or bed-mounted device) to measure your night-time scratching?
   - Yes
   - No
   - I don’t know
6. Which uses of the information collected about your night-time scratching by this technology would you find valuable? [checkboxes]

- To help me talk to my doctor about my eczema
- To help my doctor better understand my eczema
- To help me compare my eczema to those of other people
- To help me talk to friends and family about my eczema
- To help motivate myself to keep up with treatment plans
- To help researchers learn more about eczema
- To help improve my eczema
- None of the above
- Other [with freeform input]

1. Which of the following concerns you about using technology to measure your night-time scratching (during the time when you would like to sleep)? [checkboxes]
   - Appearance
   - Skin irritation
   - Sleep interference
   - Physical discomfort
   - Emotional discomfort
   - Time commitment
   - Privacy
   - None of the above
   - Other [with freeform input]

## Appendix S8. Quantitative survey: Caregiver for child patient

Introduction

In this survey, we will focus on the life and everyday experiences of your child with atopic dermatitis, which we will refer to here as eczema. We ask about your child’s eczema, their symptoms, and specifically their night-time symptoms. Night-time symptoms include those that your child may experience from the time they go to bed to sleep, during the time when they do actually sleep, and until they get out of bed after they wake up.

We will also use the terms itch and scratch, and by these we don’t mean the same thing. We will refer to “itch” as the sensation that your child has, the feeling of itchiness they perceive. We’ll use the term “scratch” as the action of scratching, meaning something your child can do to their skin. Scratching also doesn’t necessarily mean your child is using their fingers to do the scratching. It can be any physical action or motion on the skin or clothing.

Another way to think about the difference between itch and scratch is just like when you are hungry. Hunger is a feeling like itching. Eating, which is an action, is like scratching. It’s important to realize that they can happen together, or they can happen on their own. For example, you feel itchy, so you start scratching. Or, you can feel itchy and not scratch. Or, you can scratch yourself without feeling itchy.

Section 1:

In this section we’ll ask you a few questions about your child’s eczema and symptoms in general. Remember that you feel itchy, and scratching is the action.

When we ask about something happening during the day, we mean from the time you are out of bed after waking up, until the time you go to bed to sleep. When we ask about something happening at night, we mean from the time you go to bed to sleep, to the time you wake up and get out of bed.

1. Did a doctor tell you and/or your child that your child has eczema?
   - Yes
   - No
2. Based on the introduction above, which one of the following options describes ITCH?
   - The sensation, feeling on your skin
   - The action, something you do to your skin
3. How would you describe the severity of your child’s eczema:
   - Over the past 2 weeks
   - When it’s at its worst

Scale:

- - None
  - Mild
  - Moderate
  - Severe
  - Very severe

1. During the past 2 weeks, how often did you observe the following on your child?

- Issues with sleep because of your child’s eczema
- Emotional stress
- Day-time scratching
- Night-time scratching
- Red, inflamed skin
- Dry, rough, leathery or scaly patches of skin
- Oozing or crusting
- Peeling or flaking skin

Frequency selection:

- Multiple times a day
- Every day
- 5-6 days a week
- 3-4 days a week
- 1-2 days a week
- No days
- Don’t know/unsure

1. During the past 2 weeks, what is your perception of how intense your child found the following?
   - Day-time itchy skin because of your child’s eczema
   - Day-time scratching
   - Night-time itchy skin because of your child’s eczema
   - Night-time scratching

Intensity scale:

- Not at all
- Slightly
- Moderately
- Quite a bit
- Extremely

1. During the past 2 weeks, what is your perception of how burdensome your child found the following?

- Itchy skin because of your eczema
- Issues with sleep because of your eczema
- Emotional stress
- Day-time scratching
- Night-time scratching
- Red, inflamed skin
- Dry, rough, leathery or scaly patches of skin
- Oozing or crusting
- Peeling or flaking skin

Intensity selection:

- Scale with anchors for each point
  - Not at all
  - Slightly
  - Moderately
  - Quite a bit
  - Extremely

1. Rank the following from most to least burdensome for your child. Please start with the most burdensome symptom at the top.
   - Itchy skin
   - Day-time scratching
   - Night-time scratching
   - Red, inflamed skin
   - Dry, rough, leathery or scaly patches of skin
   - Oozing or crusting
   - Peeling or flaking skin
   - Issues with sleep because of your eczema
   - Emotional stress
2. To what extent does your child’s eczema limit the following aspects of their life? [5-pt scale]
   - Being spontaneous
   - Playing a sport or exercising
   - Selecting clothes
   - Eating
   - Choosing a job or career
   - Attending social or public events
   - Forming relationships with peers
   - Other [with freeform input]

- Scale
  - Not at all
  - Slightly
  - Moderately
  - Quite a bit
  - Extremely

Section 2:

In this section we’ll ask you a few questions about your child’s eczema and symptoms in general. Remember that your child feels itchy, and scratching is the action.

When we ask about something happening during the day, we mean from the time your child is out of bed after waking up, until the time they go to bed to sleep. When we ask about something happening at night, we mean from the time your child goes to bed to sleep, to the time they wake up and get out of bed.

1. During the past 2 weeks, how often did you observe your child scratching during the day (during the time when they would like to be awake)?
   - Multiple times a day
   - Every day
   - 5-6 days a week
   - 3-4 days a week
   - 1-2 days a week
   - No days
   - Don’t know/unsure
2. How aware are you of your child’s night-time scratching?

- Not at all
- Slightly
- Moderately
- Quite a bit
- Extremely

1. During the past 2 weeks, how often did you observe your child scratching at night (during the time when they would like to sleep)?
   - Multiple times a night
   - Every night
   - 5-6 nights a week
   - 3-4 nights a week
   - 1-2 nights a week
   - No nights
   - Don’t know/unsure
2. During the past 2 weeks, how often did your child wake up with the following signs that weren’t there when they went to bed?
   - Skin flakes
   - Skin cuts
   - Blood on sheets or skin
   - Open wounds or torn skin
   - Scratches on skin/scratch marks
   - Crusting
   - Oozing
   - Irritated or red skin
   - Thickening of skin

Frequency

- - Multiple times a night
  - Every night
  - 5-6 nights a week
  - 3-4 nights a week
  - 1-2 nights a week
  - No nights
  - Don’t know/unsure

1. During the past 2 weeks, how often did your child’s night-time scratching affect your child’s sleep?
   - Every night
   - 5-6 nights a week
   - 3-4 nights a week
   - 1-2 nights a week
   - No nights
   - Don’t know/unsure
2. During the past 2 weeks, on average, how many hours of sleep did your child lose because of their night-time scratching? [0-12 hour sliding scale]
3. During the past 2 weeks, how often has your child’s night-time scratching (during the time when they would like to sleep) impacted each of the following aspects of your child’s life?
   - Not sleeping at all at night
   - Not feeling rested in the morning, feeling tired during the day
   - Not being able to focus during the day
   - Lowered productivity at work or school
   - Missing work or school completely
   - Missing out on social life and activities with family, friends, neighbors, etc.
   - Not being able to take vacations or travel
   - Feeling depressed or anxious during the day
   - Less overall happiness
   - Seeking a therapy or a specialist to help with sleep issues
   - None of the above
   - Other [with freeform input]

Frequency selection:

- Every day
- 5-6 days a week
- 3-4 days a week
- 1-2 days a week
- No days
- Don’t know/unsure

1. Does your child do any of the following to prevent or reduce their night-time scratching (during the time when they would like to sleep)? [checkboxes]
   - Wear special clothes (e.g., long-sleeved or made of specific material)
   - Wrap hands or use gloves for the time you sleep
   - Shower before going to sleep
   - Apply creams, lotions or topical medications before sleep
   - Take bleach baths or use wet wraps before sleep
   - Take oral eczema medications before sleep
   - Take over-the-counter sleep medication(s)
   - Take prescription sleep medication(s)
   - Practice meditation or focused breathing
   - Lower the temperature in the room where you sleep
   - Go to the doctor to change their treatment regimen
   - None of the above
   - Other [with freeform input]

The following questions will ask about how your child’s eczema has impacted you.

1. During the past 2 weeks, how burdensome did you find the following?

- Your child’s itchy skin because of their eczema
- Your child’s issues with their sleep because of their eczema
- Your child’s emotional stress
- Your child’s day-time scratching
- Your child’s night-time scratching
- Your child’s red, inflamed skin
- Your child’s dry, rough, leathery or scaly patches of skin
- Your child’s oozing or crusting
- Your child’s peeling or flaking skin

Intensity selection:

- Scale with anchors for each point
  - Not at all
  - Slightly
  - Moderately
  - Quite a bit
  - Extremely

1. Rank the following from most to least burdensome for you about your child’s eczema. Please start with the most burdensome symptom at the top.
   - Your child’s itchy skin because of their eczema
   - Your child’s issues with their sleep because of their eczema
   - Your child’s emotional stress
   - Your child’s day-time scratching
   - Your child’s night-time scratching
   - Your child’s red, inflamed skin
   - Your child’s dry, rough, leathery or scaly patches of skin
   - Your child’s oozing or crusting
   - Your child’s peeling or flaking skin
2. During the past 2 weeks, how often did your child’s night-time scratching affect your sleep?
   - Every night
   - 5-6 nights a week
   - 3-4 nights a week
   - 1-2 nights a week
   - No nights
   - Don’t know/unsure
3. During the past 2 weeks, on average, how many hours of sleep did you lose because of your child’s night-time scratching? [0-12 hour sliding scale]
4. During the past 2 weeks, how often has your child’s night-time scratching (during the time when they would like to sleep) impacted each of the following aspects of your life?
   - Not sleeping at all at night
   - Not feeling rested in the morning, feeling tired during the day
   - Not being able to focus during the day
   - Lowered productivity at work or school
   - Missing work or school
   - Missing out on social life and activities with family, friends, neighbors, etc.
   - Not being able to take vacations or travel
   - Feeling depressed or anxious during the day
   - Less overall happiness
   - Seeking a therapy or a specialist to help with sleep issues
   - Impacted intimacy in relationships
   - None of the above
   - Other [with freeform input]

Frequency selection:

- - Every day
  - 5-6 days a week
  - 3-4 days a week
  - 1-2 days a week
  - No days
  - Don’t know/unsure

1. How important is it that an eczema treatment lessens your child’s night-time scratching (during the time when they would like to sleep)?
   - Not important at all
   - Slightly important
   - Moderately important
   - Quite a bit important
   - Extremely important
2. What improvements connected to your child’s night-time scratching (during the time when they would like to sleep) are most important to you? [checkboxes]
   - My child spending less total time scratching during the night
   - My child having fewer scratching episodes during the night
   - Reducing how hard my child scratches at night
   - My child waking less often during the night due to scratching
   - My child having less new skin damage when they wake up
   - My child having better sleep quality in general
   - My child falling asleep more easily
   - None of the above
   - Other [with freeform input]
3. How valuable would it be to measure your child’s night-time scratching (during the time when they would like to sleep)?
   - Not at all
   - Slightly
   - Moderately
   - Quite a bit
   - Extremely
4. Would you be willing to have your child use technology (for example, a wearable device or bed-mounted device) to measure their night-time scratching?
   - Yes
   - No
   - I don’t know
5. How would you use the information collected about your child’s night-time scratching by this technology? [checkboxes]

- To help me talk to my child’s doctor about their eczema
- To help my doctor better understand my child’s eczema
- To help me compare my child’s eczema to those of other children
- To help me talk to friends and family about my child’s eczema
- To help motivate myself and my child to keep up with treatment plans
- To help improve my child’s eczema
- To help researchers learn more about eczema
- None of the above
- Other [with freeform input]

1. Which of the following concerns you about using technology to measure your child’s night-time scratching (during the time when they would like to sleep)? [checkboxes]
   - Appearance
   - Skin irritation
   - Sleep interference
   - Physical discomfort
   - Emotional discomfort
   - Time commitment
   - Privacy
   - None of the above
   - Other [with freeform input]

## Appendix S9: Samples of verbatim responses from the participants

Adults with atopic dermatitis:

“*I have to double think what I wear, what I eat. My intake on beverages, fluids. Mentally I have to be very aware if my depression has been triggered, why has it been triggered. I have more awareness but some other people probably find it burdensome to have to double think or triple think.” ID1215. Female, 25-34. Severe.*

*“With eczema, or again, what I believe to have been severe eczema, it definitely affects every aspect of your life, whether you want it to or not. It’s the first thing you think of immediately when you wake up in the morning, because the first thing you think of is your skin, and you wonder, ‘What can I accomplish today? What can I get done?’ Like, first of all it’s like, okay, let me see, can I even get out of bed without my skin cracking?’“ ID1204. Female, 35-50. Moderate.*

*“Oh yes, plenty of things, I mean I think it’s affected my career choice. Well, you know, I chose not to be a teacher-, I chose not to be more, you know, in those like front-line facing roles because it involves people seeing my skin and having to kind of be on a stage all the time or the feeling of being stage. And it means I couldn’t be a chemist because you have to work with chemicals, I couldn’t beat doctor because you have to, you know, use hand sanitiser all the time and wear latex gloves and all of that.” ID1209. Female, 35-50. Moderate.*

*“I think one of the biggest things with having eczema is losing spontaneity with different activities. So, if it is really windy outside, I might not want to exercise outdoors or go for a walk, because I know that the wind will make my eczema feel worse. Like I said, I experience eczema on my face, so I get it around my mouth and around my eyes.” ID1207. Female, 18-24. Mild.*

*“The most frustrating thing is that I can’t always control the itch. Applying lotion or trying to avoid scratching, those things don’t actually help the experience, they just avoid damage to my skin. You know? So, when I would have more severe flare-ups, like when I was in high school, I would wake up with blood under my fingernails because I’d been scratching in my sleep. And so I think the fact that there’s no magic cure to just make the itchiness go away is the most frustrating part, because it definitely interferes with my ability to focus, as I’m always thinking about my skin when I’m itchy.” ID1207. Female, 18-24. Moderate.*

*“Yes, the emotional impact ... definitely get depressed thoughts and episodes and 100% more anxiety because I’m just hyper aware of my environment and what can possibly trigger it.” ID1211. Female, 18-24. Mild.*

*“For a while I felt very, very depressed. Most of the time in my life when I have a problem, you know, I feel like I’ve got good resources, I can reach out to somebody, I can do different things. And I felt like with eczema, everything I was trying was just resulting in, like, another dead-end, or a lot of doctor appointments that weren’t really getting me anywhere. I was very anxious all the time, I just felt like I wanted to cry, in fact I did cry a lot. I got to the point where I didn’t really want to go out very much during the day, I didn’t really want to go to work, I didn’t want to go out with friends after work. I just felt like, you know, trying to sleep it away. And then I got so frustrated, because even when I tried to sleep, then I would keep waking up with the intense urge to scratch.” ID1206. Male, 35-50. Mild.*

*“I’d say for me probably the number one thing that caused me so much grief back in 2018 and 2019 was the itchiness, just the feeling that I had, it felt like I had little ants crawling all over my skin. I literally wanted to just scratch my skin away, it was so frustrating. So, itchiness was definitely the number one thing, but probably the other symptoms that really bothered me, one was sometimes my skin felt very hot, I felt like I had just gotten out of, like, a hot tub or spa, or something like that. And then the third thing that bothered me a lot was sometimes my skin felt very tight, it felt like somebody was pinching me constantly.” ID1206. Male, 35-50. Mild*

*“I do scratch in the middle of the night and it sometimes wakes me up. When it’s bad maybe, like, four times. When it’s good maybe, like, once. Sometimes I think it’s more also having trouble falling asleep because I tend to be itchier at night.” ID1209. Female, 35-50. Moderate.*

*“I think it gets worse at night, just because if I end up falling asleep I do scratch in my sleep and sometimes I’m not consciously aware of it. Sometimes I’ll be half awake and, kind of, sense that I’m moving, you know, like, the scratching motions, but I can’t really stop myself from the action.” ID1212. Female, 25-34. Severe.*

*“Yes, I think it’s just difficult, and it’s difficult to even explain it, if people haven’t experienced it, because it has such an impact on your mental health and everything that you do during the day. So, it’s kind of your entire life, it’s all-encompassing. So, it’s a difficult thing to live with. It’s not easy. So, I think people that say, ‘It’s just skin, don’t worry about it,’ tend to trigger most of us, because it’s really not easy to live in an itchy skin suit every day.” ID1210. Female, 25-34. Severe.*

*“It probably depends on stress level and also my environment, like, maybe it was particularly hot that night, or maybe there’s dust in the room, or maybe my husband snored that night so then I woke up, so then I would start scratching because I woke up.” ID1209. Female, 35-50. Moderate.*

*“Definitely scratch marks, luckily I haven’t been bleeding from my eczema so much recently. But yes, definitely I notice it on my arms the most, when I wake up, if I’ve been scratching in the night, I’ll have raw skin or inflamed skin. Yes, the signs or if I woke up in the middle of the night. Probably four or five times a week.” ID1201. Female, 25-34. Moderate.*

*“I mean, there’s always signs of the blood on the sheets, the flakes, oh my god, so many flakes everywhere, from your sheets, in your hair, on the floor. Tears in the sheets, that’s the worst part, because you’re like, ‘Oh, great, another set of sheets that I went through that I’ve torn up again, that’s really expensive.’ So, that because you’re constantly moving and itching, your legs are itching themselves at night, and then your skin is all torn up or you’re really sore, your open wounds are bleeding, like, oh my god, it’s quite a fiasco, yes.” ID1204. Female, 35-50. Moderate.*

*“Oh man, you know, the quality of life would be so much different (with effective treatment). You know, unfortunately, like, at this current moment I don’t have major medical insurance so everything I use for my eczema regiment comes out of pocket. And so I’m very limited on the types, very, very limited on the types of treatments that I can get.” ID1214. Female, 35-50. Moderate.*

*“I wouldn’t mind honestly (wearing a tool). I wouldn’t mind, anything that gets us closer to figuring out a cure or some type of treatment or just making it better, even if it’s not for me, for someone else, or a child who’s going through what I had experienced when I was that age and I wasn’t sleeping because of it and all of that, definitely yes, 100%. So, yes, I would definitely be up to doing that, if that’s something that’s possible, yes.” ID1202. Female, 18-24. Mild.*

Adult caregiver/spouse/partner of an adult with atopic dermatitis

*“Well, I wake up every morning at 5:30 in the morning for work and I do a lot of east coast hours. And if my wife has woken up a couple of times from itching at night and it kept me awake, so I only got about 2 or three hours of sleep. And then I’m working for nine or ten hours.” ID1203. Male, 35-50. Mild.*

*“It does because like she feels emotionally distressed we end up sometimes disagreeing and fighting and like just, in general, she wants me to spend more time with her than anybody so it just turns around to me not being able to be as social as I used to be before.” ID1213. Male, 35-50. Severe.*

*“Yes, at least I’m more conscious of waking up and seeing that her arms are red or there’s a little bit of blood on the inside of the elbow and I haven’t seen that during the day.” ID1201. Male, 25-34. Moderate.*

*“I just think, like, the frequency (of scratching) is more. And it may result in her waking up more to scratch, or something, kind of, like that.” ID1209. Male, 35-50. Severe.*

*(Importance of treatment for nocturnal scratching) “Definitely, I would get better sleep, she would get better sleep. Then we would functionally be much healthier and enjoy our lives more.” ID1201. Male, 25-34. Moderate.*

“*She’ll have more energy. She’ll be able to get up early, definitely get up early from the time that she wakes up in the morning to do whatever she needs to do to tackle her day, to get herself ready. That time is kind of rare, to get that time, when you’re not sleeping properly. That alone would be a great improvement.” ID1215. Male, 25-34. Severe.*

Children with atopic dermatitis

“*Okay, maybe some of my friends think that they catch the affliction. Yes, because they have already chosen to just stay away from me.” ID3412. Male, 13-17. Severe.*

*“If I want to play something but I can’t because of my eczema or something? Well, sometimes with all my owies I can’t go swimming, and I don’t like that. And I love swimming.” ID3404. Male, 6-9. Moderate.*

*“Sometimes I want to hang out with friends or maybe family but then I’m having a hard time because of the itching, scratching and maybe the way I look so I have to avoid people because they end up staring.” ID34 11. Female, 13-17. Severe.*

*“Yes. I want to do, like, volleyball and soccer, but soccer, you sweat a lot and the sweat also affects my skin. And it gets, like, really hot outside. In volleyball, like, they do it outside, so I can’t do that, and I would like to try soccer and volleyball.” ID34 01. Female, 6-9. Moderate.*

*“Because it prevents me from participating in a lot of things and also it makes me feel emotional at times. I cry from the pain.” ID34 11. Female, 13-17. Severe.*

*“Yes, I feel like I scratch more at night than I do at day.” ID34 02. Male, 6-9. Mild.*

*“Yes, because during the day I’m more awake and I know what I’m doing. Whereas, if I’m half awake, sleeping, itching my skin, I won’t be able to stop myself because I’m not aware that I’m fully doing it. There’s definitely more control during the day than there is at night.” ID34 09. Female, 13-17. Severe.*

*“Little worse at nights. Because during the day I’m doing my stuff, so sometimes I don’t itch that much and then when at night I’m not really doing anything, it’ll just start itching.” ID34 08. Male, 13-17. Moderate.*

“*It, like, itches and then after I start scratching it I can’t stop scratching it and then it hurts really bad. Yes the winter is worse. it’s drier so it, like, makes my skin drier and then it itches more. ID3410. Female, 13-17. Moderate.*

*“Yes, I wake up and disturb my daddy to take some ointments.” ID34. Male, 13-17. Severe.*

*“Yes. Sometimes, like, it takes me so long to fall asleep, and then when I finally fall asleep, I wake up because I’m really, really itchy. My mum, she has an Apple Watch and, like, sometimes I have to sleep with her. One time I slept with her, I checked her Apple Watch and it said it was three in the morning, so I didn’t want to take a shower then because my mum was already asleep, in a deeper sleep. I tapped her, but she, like, wouldn’t wake up. So, I got up. I put some cream on and I tried to fall asleep. I fell asleep at, like, four, I think, because of my itchiness and I was very tired the next day and very itchy.” ID34 01. Female, 6-9. Moderate.*

*“A good night is when I have a cold blanket, because that always helps me go to bed. When I feel too hot, it makes me have a bad night.” ID3416. Male, 10-12. Mild.*

*“I know lately I don’t have that much energy, and I get really moody on the nights where I can’t fall asleep that quickly or stay asleep, because the eczema is itchy. I’d definitely be able to focus more as well, and not be so exhausted during the day.” ID34 09. Female, 13-17. Severe.*

*“Yes, it has an impact, because maybe during the day I’ll have wounds which will make me uncomfortable maybe sometimes when I walk, my joints are in pain. The immediate impact that I’m actually separated from my friends, I feel lonely at times. It’s just a bad experience.” D34 12. Male, 13-17. Severe.*

*“I know I’ll be really exhausted, so I won’t really want to pay attention in school or focus on school, I’d rather actually be taking a nap instead. For choir, sometimes I won’t sing and I’ll pay attention but I just won’t actually sing because I don’t have the energy to.” ID34 09. Female, 13-17. Severe.*

*“Sometimes, there are skin flakes, and then when I wake up in the morning and I make my bed, there’s sometimes a little bit of blood on the bed. The flakiness happens quite a bit, but the blood doesn’t happen a lot. Sometimes it bleeds.” ID34 16. Male, 10-12. Mild.*

*“Sometimes I’ll just wake up and have random scars from scratching and I didn’t even know I scratched.” ID34 08. Male, 13-17. Moderate.*

*“I do the Aron regimen. Normally I’ll do my medicine and then I will let it soak in, I go on my phone, and then normally from there I’ll fall asleep if I don’t itch in between, but most times I do.” ID34 09. Female, 13-17. Severe.*

*“I would (wear a tool). To, like, see if I’ve been scratching more than I realised, to see whether my eczema is doing better because I can see where-, today’s is and can see how much I scratched, it might be better than today’s.” ID34 07. Female, 6-9. Moderate.*

*“Yes. It (a tool) would show how much I’m actually awake and itching during the night and trying to pinpoint when it’s happening or if it’s happening at certain times of the night, and maybe pinpoint why it’s happening. ID34 09. Female, 13-17. Severe.*

Adult caregiver of a child with atopic dermatitis

*“Okay, I may say since she was a kid, this condition has really affected her daily life, including us, not just hers. You know, maybe just measuring on her, some of the challenges she has really faced is about stress, actually, dealing with stress, depression and how to deal with the emotional damage from friends. This condition, not so many people do know that it’s not contagious, so when some other kids look at her, they’re like-, then her skin is, like, flaring, she’s scratching her skin, her hands all over, they cannot interact with her. So, this has really affected her mental wellbeing and, of course, even social interaction, yes.” ID34 11 - (SEV-F13-17) - (DAD-M25-34)*

*“Her life is impacted daily by eczema because she has to create extra time to make sure she does self care and her medications, so that she does not flare any worse than normal. Because if she flares badly, then she has no life. She’s sitting in bed, she can’t wear clothes, she’s being medicated every 2 hours, and then ice packs in between. She’s miserable, and depressed, and in a lot of pain. So, if you don’t keep it under control, it affects every aspect of your daily life. And, by trying to control it, can do the same thing, you know? It affects how she goes to school, when she has to take breaks to put medication on, how long she can sit and study before she has to take a break to put medication on. I mean, every aspect. What she eats, how she eats it, because, like oranges, if she has too much citrus it’ll break her out and make her itchy. What shoes she has to wear, what clothes she wears can exacerbate it. Anything environmental can cause issues. But, as she said, just everything.” ID34 09 - (SEV-F13-17) - (MUM-F35-50)*

*“Well, school has been one of the hardest ones because like she was saying, when she doesn’t sleep good at night, she’s not fully aware in the morning. Like, she’s tired, it’s hard for her to concentrate at school when she starts getting anxious because she doesn’t get something she starts scratching her hands, so it’s hard for her to write because her hands are itchy or they hurt. Because she scratched them so much that now she can’t take the pencil.” ID34 01 - (MOD-F6-9) - (MUM-F35-50)*

*“The scratching, obviously, or the itching that leads to the scratching which leads to pain has the biggest impact on her life. And then second would be because of that you have the lack of sleep which causes deficits, you know, the attention deficit, the sleep deficit, which causes your body not to function properly.” ID34 09 - (SEV-F13-17) - (MUM-F35-50)*

*“I would probably say it’s the scratch. Like, he spends so much time doing it. I mean, I think it’s his normal, unfortunately. I think he would say, if he could take the itch away that would probably be the number one thing.” ID34 06 - (SEV-M10-12) - (MUM-F35-50)*

*“Oh, on my big life, it’s just that I would like to have more company of him, to know what he thinks. You know, to make sure that he’s developing in the right way. But, know that he’s keeping to himself. It also makes you as a parent think twice, you know. I think, I wish it happened to me but not to him. Something of this thought.” ID34 12 –(Sev-M13-17 – (Dad-M25-34).*

*“The biggest impact I can talk about is stress. Living with stress. All those tough, sleepless nights, you are not going to sleep, the kid wakes up at midnight starts crying (inaudible 37.06), scratching everywhere, then you just. That is happening. What are you going to do? Living with that becomes hectic.” ID34 11 - (SEV-F13-17) - (DAD-M25-34)*

*“Sometimes I don’t do my own self care. You know, the laundry piles up, or, like, your housework. It’s not like you can deep clean and go through, like, cupboards and cupboards of stuff because you just, you know, like, your binge and purge cycles just for a living. Like, you know, taking care of the sheds or taking care of a window that needs to be fixed. A lot of that has to be put on hold.” ID34 09 -(Sev-F13-17) – (Mum-F35-50).*

*“Yes, I worry constantly, I’m always on the look out for something different, I’m constantly thinking about it, reorganising, reformulating different game plans. For treating it, like, finding different things that we can incorporate possibly into his diet or into his lifestyle or take away or whatever in order to make things work better.” ID34 08 - (MOD-M13-17) - (MUM-F51-64)*

*“I would say another impact would be choosing activities. What we’re going to do as a family. Sometimes we choose not to do something because we know that it’s going to affect his eczema and flare it up and he’s just not going to enjoy his time.” ID34 03 - (MOD-M6-9) - (MUM-F35-50)*

*“Bloody sheets, I would say is still a couple of times a week, for sure. Redness, every night. Waking up with an oozing spot, maybe once or twice a week nowadays. The flaking is pretty daily, as well.” ID34 04 - (MOD-M6-9) - (MUM-F35-50)*

*“Usually, the back of his legs will be red, like, they’ll be redder, which, for the most part we know when he’s scratched because it’ll have an inflamed look to it. And like you said, when he wakes up in the morning, that’s the first thing he’s doing. To stretch, he’ll be scratching. Yes, I would say every night.” ID34 03 - (MOD-M6-9) - (MUM-F35-50)*

*“Night-time has always been worse. I don’t know if it’s because-, I don’t know why. I’m not really sure medically why it’s worse at night. It may be because he doesn’t have other things to occupy his mind, maybe, like he does during the day. Sometimes it’s maybe it’s just a habit, he’s been doing it for so long. I know he does itch, and just depending on how bad his allergies are, and what we’ve been doing that day could affect the night time also. Yes, night-time has always been bad.” ID34 06 - (SEV-M10-12) - (MUM-F35-50)*

*“It (waking in the night) happens often. Like, three, four times a night. Yes. I mean, it’s not every night, but when it happens during the night, she then wakes as many as three, four times, yes. When she has flares, like, when she is fine, she is clear, she will sleep better.” ID34 01 - (MOD-F6-9) - (MUM-F35-50)*

*“I would say it probably takes him at least a half hour to 45 minutes (to go back to sleep). If not longer, to go to sleep. Because like I said, you know, when he wakes up sometimes, because we’ve had to move his room around in our house to be closer to us because he would get up in the middle of the night and then he would go and play video games or go turn on the TV. ID34 03 - (MOD-M6- 9) - (MUM-F35-50)*

*“It requires much of my time taking care of him, during the night. Yes. I normally wake up, check on him. Yes. Sometimes I don’t sleep.” ID34 14 - (SEV-M13-17) - (DAD-M25-34)*

*“Yes. So he’s a very much a creature of habit. So the room is always cool and he has his special sheets and blanket that he uses. Part of it is just placebo effect, he feels like if he has all of the above that it’ll be a good night. So we have his special sheets and a very thin blanket, we use a Dyson fan.” ID34 16 - (MILD-M10-12) - (MUM-F35-50)*

*“Yes. But currently, we try to control the air quality and the air humidity in his room to help with the allergy aspect of that. He also has to have 100% cotton bedding that’s washed weekly. And, he has a very, kind of, routine bedtime routine. So he, you know, brushes his teeth, lays in bed. Whenever, you know, his dad or I usually stays in the room until he falls asleep because of the itching. So, we’re trying to help, kind of, calm it until he can go to sleep.” ID34 04 - (MOD-M6-9) - (MUM-F35-50)*

*“If we could find a better treatment for her I think it would be wonderful but we, kind of, treat as it happens instead of having a preventative thing and if there was a way to prevent it from happening, I think that would be amazing. Yes I think it would give her better quality of life if she slept better. Mentally it would, I don’t think any other. It would help me to not worry about her so much.” ID34 10 - (MOD-F13-17) - (MUM-F35-50)*

*“Absolutely (treatment would be important). I mean, when he’s not getting a good night’s sleep, and I’m not even going to talk about myself, but for him, he’s had times at school where he falls asleep. I think the teachers assume that I’m letting him stay up late, or something, or you know, he’s not getting a good night’s sleep. I put him to bed at a reasonable hour, where he should be getting ten hours of sleep, but when he’s up scratching frequently it affects him at school the next day, where he’s tired and is unable to maybe learn all the lessons like he should be, because he isn’t adequately sleeping. As a young child, he’s just developing and having to meet all these milestones in school to progress on to the next grade. It’s been a struggle when he’s not getting a full eight to ten hours of sleep every night.” ID34 06 - (SEV-M10-12) - (MUM-F35-50)*

## Appendix S10: Selected survey data tables

Table S10.1.: Atopic dermatitis impacts & effects on life, n (%)


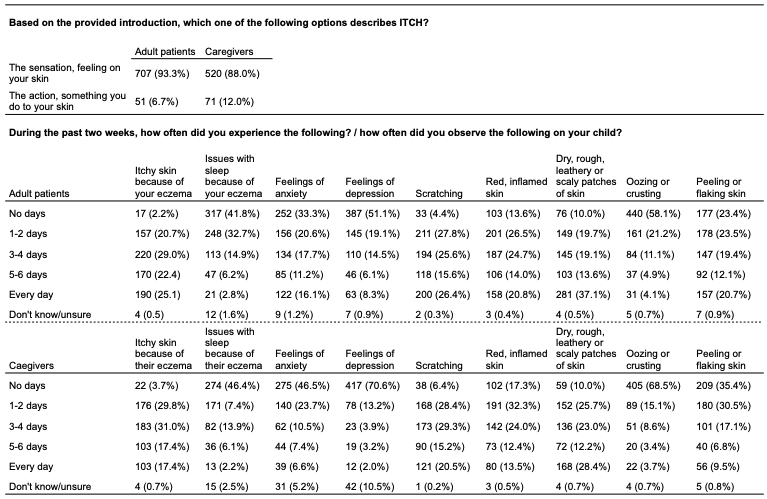


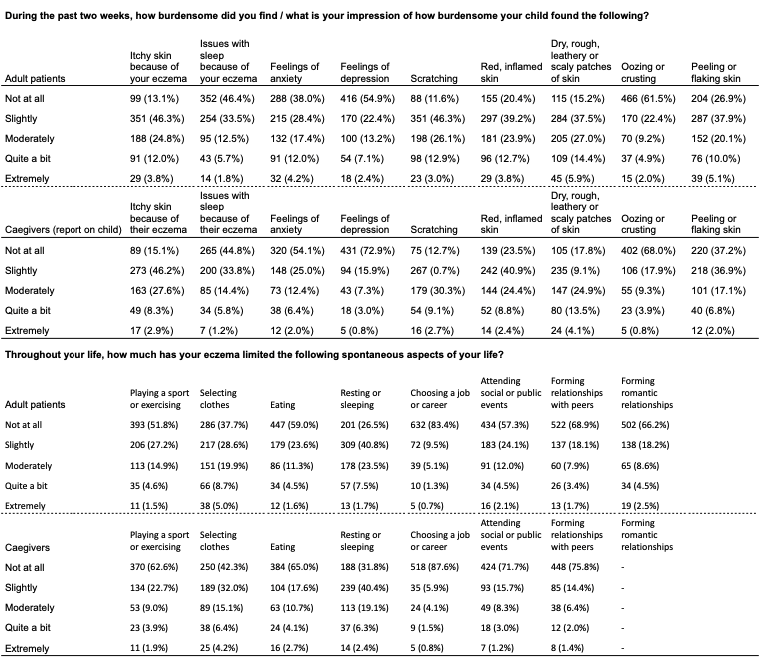


Table S10.2.: Nocturnal scratching, n (%)


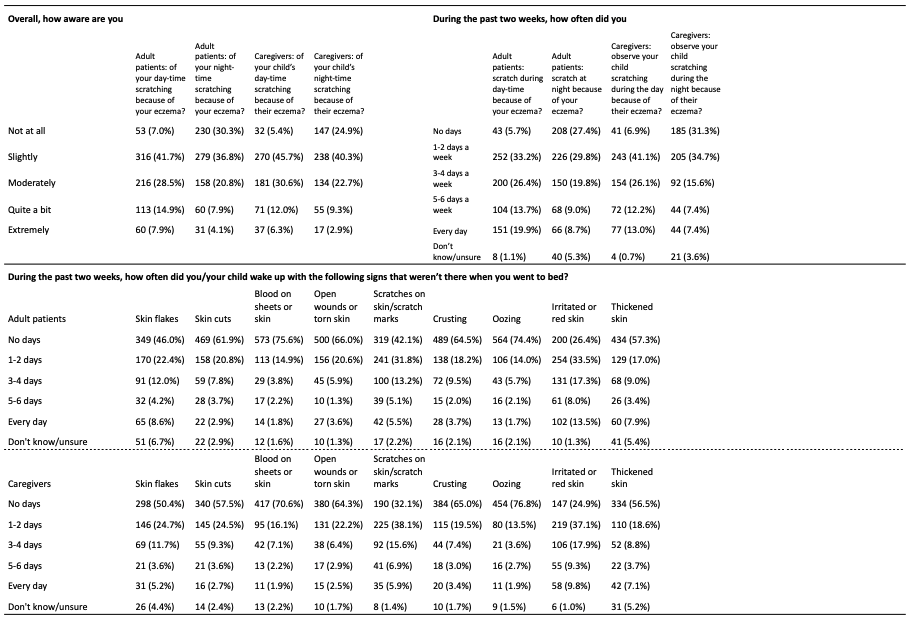


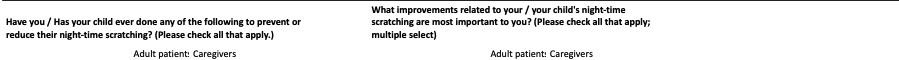


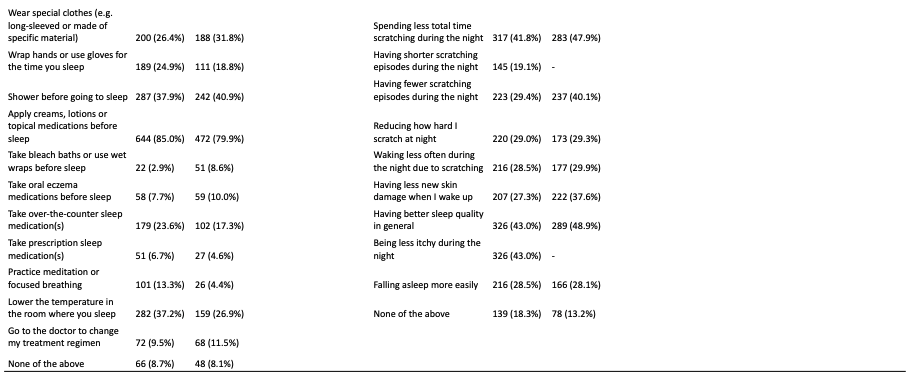

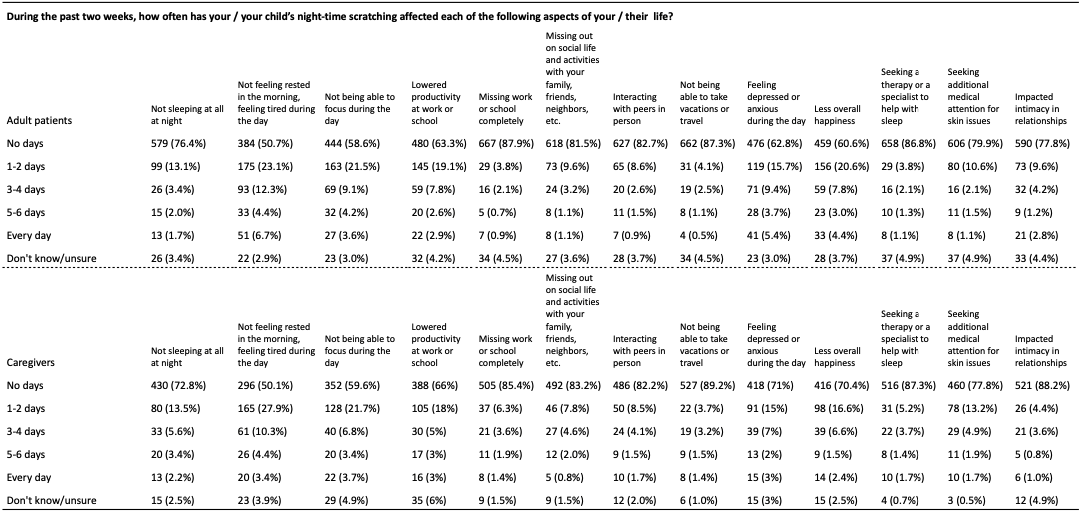

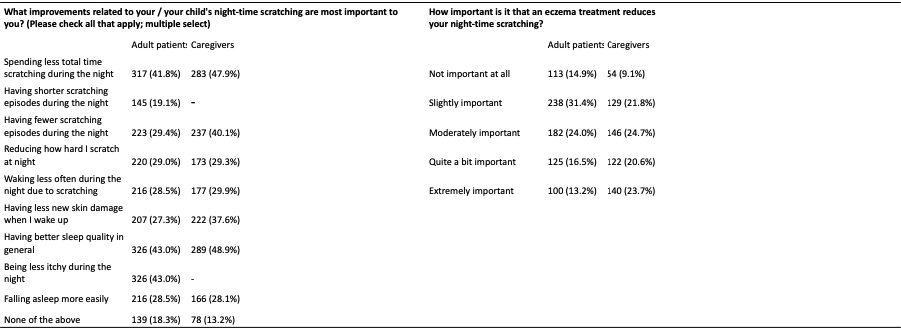


Table S10.3.: Hours of sleep lost per group
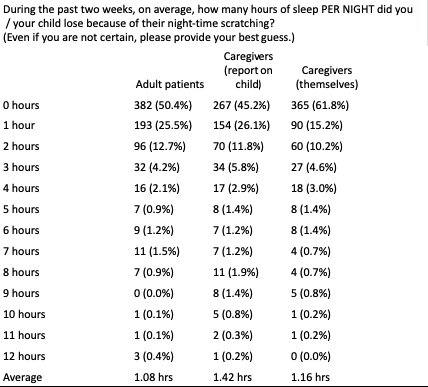


Table S10.4.: Measurement of Nocturnal Scratch and Use of Digital Technologies, n (%)


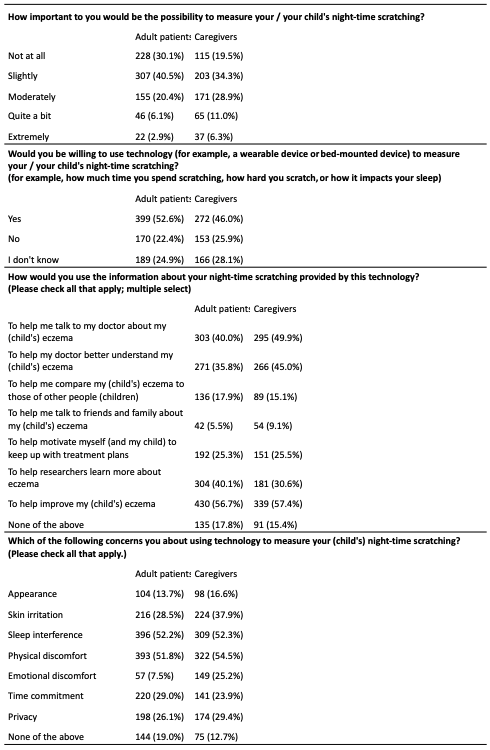

Supplement: Supplementary file 1 — Supporting Information S1 [file SKI2-3-e262-s001.docx]
